# Proteomic profiling based classification of CLL provides prognostication for modern therapy and identifies novel therapeutic targets

**DOI:** 10.1038/s41408-022-00623-7

**Published:** 2022-03-17

**Authors:** Ti’ara L. Griffen, Fieke W. Hoff, Yihua Qiu, James W. Lillard, Alessandra Ferrajoli, Philip Thompson, Endurance Toro, Kevin Ruiz, Jan Burger, William Wierda, Steven M. Kornblau

**Affiliations:** 1 Department of Microbiology, Biochemistry, and Immunology, Morehouse School of Medicine, Atlanta, GA USA; 2 Department of Internal Medicine, University of Texas Southwestern Medical Center, Dallas, TX USA; 3 Department of Leukemia, The University of Texas MD Anderson Cancer Center, Houston, TX USA; 4 Department of Medicine, University of Central Florida College of Medicine, Orlando, FL USA

**Keywords:** Cancer genomics, Chronic lymphocytic leukaemia

## Abstract

Protein expression for 384 total and post-translationally modified proteins was assessed in 871 CLL and MSBL patients and was integrated with clinical data to identify strategies for improving diagnostics and therapy, making this the largest CLL proteomics study to date. Proteomics identified six recurrent signatures that were highly prognostic of survival and time to first or second treatment at three levels: individual proteins, when grouped into 40 functionally related groups (PFGs), and systemically in signatures (SGs). A novel SG characterized by hairy cell leukemia like proteomics but poor therapy response was discovered. SG membership superseded other prognostic factors (Rai Staging, IGHV Status) and were prognostic for response to modern (BTK inhibition) and older CLL therapies. SGs and PFGs membership provided novel drug targets and defined optimal candidates for Watch and Wait vs. early intervention. Collectively proteomics demonstrates promise for improving classification, therapeutic strategy selection, and identifying novel therapeutic targets.

## Introduction

Chronic Lymphocytic Leukemia (CLL), the most common adult leukemia, is an indolent B-cell malignancy predominantly diagnosed in older people [1]. Several molecular factors influence CLL biology and prognosis: Immunoglobulin Heavy Variable mutation status (IGHV, Unmutated U-/Mutated M-CLL), cytogenetic aberrations (deletions 17p, 11q, and 13q and trisomy 12), and high expression of ZAP70 and CD38 [2–6]. The majority of CLL patients are initially observed without treatment (Watch and Wait (WaW)). CLL therapy has significantly evolved as new treatment modalities were introduced [7]. Initially cytotoxic therapies, predominantly alkylating agents (1960s), purine analogs alone (1970s–80s), or a combination of the two (1990s) were used. Treatment markedly changed with the development of the anti CD20 monoclonal antibody rituximab (1998) used alone or as combined chemoimmunotherapies (fludarabine, cyclophosphamide, and rituximab (FCR)). These modalities dominated until 2014 with the development of therapies inhibiting B-cell receptor signaling by targeting BTK (Ibrutinib, Acalabrutinib), or PI3K (Idelalisib, Duvelisib) or apoptosis regulation by targeting BCL2 (Venetoclax) [8–13]. The decision on when to treat and what therapy to utilize is determined based on the presence of symptoms, patient age and comorbidities, and certain prognostic markers such as the 17 P deletion/TP53 or IGHV-mutation status. Currently, when therapy is warranted, most patients receive a BTK inhibitor with or without an antibody (Rituximab/Obinutuzumab) or combination of anti-CD20 mAb and Venetoclax [14]. Younger patients with M-CLL may still be offered chemoimmunotherapy, FCR.

The availability of highly effective therapy and the concurrent increased molecular understanding of CLL requires the re-evaluation of treatment paradigms. Are there high-risk patients who should be treated upfront, because their WaW period can be predicted to be brief, and others for whom WaW remains appropriate? What criteria can we use to select these patients? Similarly, with many potential therapies to select from, can we match individual patients to specific molecular characteristics to rationally select therapy and improve outcomes? Traditional prognostic factors were successful at stratifying patient risk groups for survival and treatment strategies, however, in the modern targeted therapy era some are no longer relevant (i.e., ZAP70). Furthermore, many different molecular events can co-occur in the same patient complicating therapy allocation based on the presence/absence of a single genetic event. There is therefore a need for a means to recognize the integrated consequence of all the internal molecular events and the external environment influences that affect CLL cell biology, for each patient, in order to provide prognostication for therapy need (predict WaW interval, TTFT) and response to different therapies.

Since the end consequence of all genetic, epigenetic, and environmental influences on the cell occurs at the protein level we hypothesize that proteomic analysis of CLL can provide this missing information. Currently, several methods exist to quantify protein expression levels: immunohistochemistry (IHC), ELISA, flow cytometry, Mass spectrometry, and protein arrays. Antibody-based techniques (i.e., IHC, flow cytometry, ELISA, reverse phase protein array (RPPA)) have increased sensitivity as antibodies are designed to solely bind to certain proteins. However, ELISA tests require a large amount of patient sample, and like IHC, is only capable of quantifying a single protein in one sample at a time, making these less applicable for large-scale studies and precluding unbiased analysis of the proteome. Mass spectrometry could provide data on the complete proteome, but this methodology is not practical for the rapid analysis of large numbers of patients. RPPA is a method capable of a collective simultaneous assessment of a large number of total and post-translationally modified proteins (PTMs) in a large number of samples, requiring only a small amount of patient sample. This methodology, while limited to targets with validated antibodies and therefore not “unbiased”, strikes a compromise between the technical limitations of Mass spectrometry and individual protein analysis methods. The application of proteomics methods has shown great promise for characterizing CLL biology. In particular, previous CLL proteomics studies highlighted that elderly CLL patients have deregulated inflammatory and DNA damage responses, ROS generation signaling utilization, interactions between CLL cells and their microenvironment, CLL cell immunoreactivity antigens, and recently how Trisomy 12 and IGHV status are primary drivers determinants of CLL proteomics biology [15–19]. However, the sample size of these studies makes it difficult to completely ascertain how a culmination of factors (i.e., stage, age, race, gender, mutational status) influence CLL proteomics biology.

Here, we present the largest CLL proteomics study to date which identified recurrent protein expression signatures (SG), that classify CLL and identify a novel subset, and which are strongly predictive of time to first therapy (TTFT), overall survival (OS), and display differential responsiveness to modern therapy.

## Results

### Global proteins expression in CLL

To paint a “big picture” of protein expression in CLL, 871 patient samples were printed on RPPA slides and probed with 384 validated antibodies that passed quality control criteria, which recognized total (*n* = 302) or post-translationally modified (*n* = 82) proteins. Biases in the data based on sample collection intervals (diagnosis to sample), organ (PB or BM), processing of fresh vs. cryopreserved cells, treatment status prior to collection were looked for but were not observed (Supplementary Fig. S1). Additionally, clustering and differential expression of same day matched CLL BM and PB, samples found no difference.

The proteins were normalized against expression in normal CD19 + B-cell controls with expression shown in Supplementary Fig. S2. Expression was universally absent (Indigo, *n* = 14 i.e., E2F1, ITGB1, SQSTM1, SMAD5, CDC25C) or consistently very low (all sample blue, *n* = 48 i.e., SOD1, RB1, PARK7, CD74, PIK3CB) for 16% of the proteins measured. In contrast, universally high expression across all samples was uncommon only occurring in six proteins (dark red, LEF1, PXN, ZAP70, CD4, S100A4, PDCD4).

### Identifying individual proteins that are prognostic in CLL

First, we asked which proteins are individually prognostic for CLL clinical outcomes. The clinical significance of proteins was evaluated as continuous variables (Supplementary Table S1) and by splitting their expression by median, tertiles, and sextiles. As we were testing a large number of variables, we used a cutoff of false discovery rate (FDR) *p* ≤ 0.05; based on this cutoff we would expect to find 19.2 significant variables. We observed that a high number of proteins were significant for OS (*n* = 59 by median, *n* = 78 by tertile, *n* = 79 by sextile, *n* = 130 continuously), TTFT (*n* = 52 by median, *n* = 56 by tertile, *n* = 45 by sextile, *n* = 61 continuously), and TTST (*n* = 11 by median, *n* = 14 by tertile. *n* = 18 by sextile, *n* = 1 continuously) (blue proteins in Supplementary Fig. S3). The prognostic capabilities of proteins were validated using iterative Cox hazard tests (*n* = 200) on randomly split training and test data. Multiple proteins (*n* = 42 for OS, *n* = 38 for TTFT, and *n* = 12 for TTST) were prognostic in >70% of the test sets. The lists contain previously reported (i.e., H3K27Me3, MCL1, and BCL2L11) and novel (i.e., NCSTN, SGK3, HSPD1, VTCN1, TRAP1, SOD1, and TAZ) CLL prognostic proteins. This list of proteins can be used to generate hypotheses regarding what pathways CLL cells rely upon as an identifier for potential cellular vulnerability points. For example, SOD1, not previously identified as prognostic in CLL, predicted both OS and TTFT (Supplementary Fig. S4), implicating reactive oxygen species scavenging in CLL pathogenesis. We also endeavored to evaluate the clinical significance of individual proteins in the context of BTKi therapy for the 108 receiving drugs in this class. A multitude of proteins were significant for OS by median (*n* = 3), tertile (*n* = 12), sextile (*n* = 37), and continuously (*n* = 62). Three proteins (LYN, MEF2C, NUMB) were significant for all levels of stratification (Supplementary Fig. S5). These proteins could potentially serve as additional targets for inhibition or replacement in combination with BTK inhibitors.

### Most protein functional group expression patterns in CLL are leukemia-specific

Second, we wanted to collectively evaluate proteins with a shared function. Proteins were sorted into 40 protein functional groups (PFG) based on known relationships from the literature or strong correlations within the dataset. Unbiased k-means clustering of each PFG was performed utilizing a gap statistic-based algorithm (Supplementary Table S2) to identify the optimal number of clusters for each PFG, identifying 150 expression patterns overall across the 40 PFGs. Each expression pattern was defined as “Protein Cluster”. To identify which protein clusters are similar to normal CD19 controls (Supplementary Fig. S6) a linear discriminant analysis and principal component analysis was performed. Compared to CD19 normal controls overall 39% of PFG (58/150) were normal-like, and normal-like patterns were found in all but two PFG (PKC, UBQ), with 17 PFG having >1. Leukemia specific patterns were seen for all PFGs. Therefore, nearly all PFG contained both normal-like and leukemia restricted clusters.

### PFGs associated with clinical outcomes

**Fig. 1:**
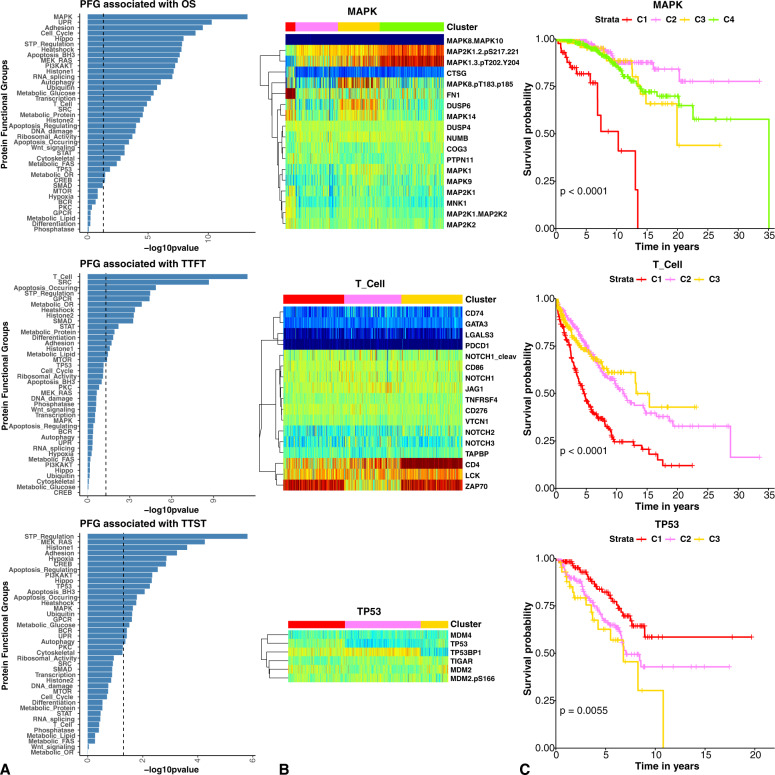
Protein functional groups associated with clinical outcomes. **A** Kaplan–Meier significance (*x*-axes) of protein functional group (*y*-axes) patient clusters are displayed in barplots. Dotted lines represent the - log10 (0.05) *p*-value cutoff. As examples, the expression patterns of the MAPK, TCell, and TP53 PFG protein members (**B**) and their Kaplan–Meier plots (**C**) are also displayed for each outcome.

We observed that outcome based on PFG were strikingly prognostic with 32 predicting OS, 17 prognostic for TTFT, and 19 predicting TTST (Fig. 1A), with most remaining significant after FDR correction (*p* < 0.05, median = 69%), markedly exceeding the two events expected by chance using the *P* < 0.05 threshold. Notably adhesion, apoptosis-occurring, apoptosis-regulating, heatshock, Histone1 (marks), Histone 2 (modifiers) and the STP-regulation PFG were prognostic for all three outcome measures. The clinical and molecular implications of the histone proteins in CLL were reported in a separate manuscript [20]. A higher percentage of PFGs were prognostic with the systems biology approach compared to analysis of individual proteins (78% vs. 19% were prognostic.) For 16 PFGs this was driven by a single cluster with a different outcome (Supplementary Fig. S7), 15 were more heterogeneous between the different PFG clusters (Supplementary Fig. S8), and nine had no differences (Supplementary Fig. S9). As an example, within the MAPK PFG (Supplemental Fig. S7, row 2, column 2) a single group (cluster 1(C1)) has adverse prognosis relative to the other three protein clusters, whereas in apoptosis-BH3 (Supplementary Fig. S8, row1, column 1) there is a stepwise decrease in survival between clusters. MAPK PFG cluster C1 (Fig. 1B, left), is distinguished by lower levels of activating phosphorylation of MAP2K1, its target MAPK1 as well as MAPK8, compared to the other three clusters which have high levels of phosphorylation. This suggests that MAPK pathway utilization is active in most cases of CLL, but, in a small subset (6%), non-utilization occurs and this may contribute to a reduction in sensitivity to therapy. This demonstrates the potential to recognize an increased level of information obtainable from analyzing proteins by systems, rather than individually.

### Integrated Metagalaxy analysis of all proteins identifies recurrent signature groups and constellations of correlated PFGs in CLL and other B/T cell derived hematological malignancies

**Fig. 2:**
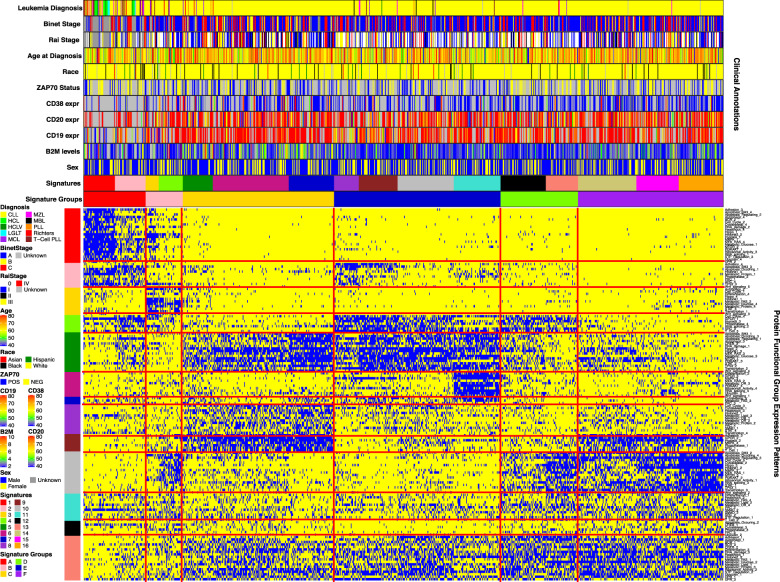
Metagalaxy heatmap of signatures, constellations, and clinical annotations. The heatmap displays the presence(blue) and absence (yellow) of 152 protein functional group expression patterns in all patients (labeled on the right). Annotations above the heatmap display signatures, signature groups, B2M levels, CD19, CD20, and CD38 expression, ZAP70 Status, age at diagnosis, staging, and diagnosis. Thirty constellations are annotated on the left side of the figure.

We next wanted to see if higher-order relationships exist between the different PFG expression patterns that would better classify, prognosticate, and discern similarities between CLL and related Mature Small B-cell Neoplasms/Leukemias (MSBL). Each patient belonged to one cluster from each of the 40 PFGs and the 150 expression patterns underwent unbiased hierarchical clustering, identifying co-occurring PFG expression patterns (constellations), and recurrent signatures formed by patients with similar patterns of constellation membership. From a total of 63 possible signature (range 9–15) and constellation (range 9–17) combinations, an optimal number (13 Constellations, 16 signatures) were determined algorithmically (Supplementary Table S3). The 16 patient signatures were further grouped into six signature groups (SG) A–F based on similarities in constellation patterns and clinical outcomes (Supplementary Fig. S10 and Fig. 2). The demographic, molecular, clinical, and response characteristics of the six signature groups are shown in Table 1.

**Table 1:** Signature group demographics and clinical information.

ALL patients	Total	A	B	C	D	E	F	*P-value*
	*N* = 871	(*N* = 84)	(*N* = 51)	(*N* = 206)	(*N* = 106)	(*N* = 227)	(*N* = 197)	
**Diagnosis**								**3.66E-44**
**CLL**	795 (91%)	48%	86%	96%	96%	95%	99%	
**HCL**	12 (1%)	14%	0%	0%	0%	0%	0%	
**HCLV**	4 (0%)	4%	0%	0%	0%	0%	0%	
**LGL-T**	4 (0%)	5%	0%	0%	0%	0%	0%	
**MBL**	4 (0%)	1%	0%	0%	0%	1%	0%	
**MCL**	12 (1%)	5%	4%	1%	1%	1%	0%	
**MZL**	12 (1%)	7%	0%	0%	2%	1%	1%	
**PLL**	4 (0%)	0%	2%	1%	0%	0%	0%	
**Richters**	8 (1%)	4%	2%	1%	1%	0%	0%	
**T-cell PLL**	16 (2%)	13%	6%	0%	0%	1%	0%	

CLL patients	Total	A	B	C	D	E	F	
	*N* = 795	(*N* = 40)	(*N* = 44)	(*N* = 198)	(*N* = 102)	(*N* = 215)	(*N* = 196)	
**Age (mean** + **SD)**	65 (±10)	63 (±8.6)	64 (±12)	66 (±9.5)	64 (±11)	63 (±11)	66 (±11)	**0.01**
**Race (772 reported)**								0.84
Asian	7 (1%)	3%	0%	1%	0%	2%	2%	
Black	33 (4%)	5%	3%	5%	3%	3%	5%	
Hispanic	22 (3%)	3%	5%	2%	3%	4%	2%	
White	710 (92%)	89%	92%	92%	94%	91%	91%	
**Gender**								0.11
Male	485 (61%)	42%	55%	63%	68%	61%	60%	
**Binet stage (784 reported)**								**0.0002**
A	478 (61%)	47%	73%	49%	71%	68%	60%	
B	71 (9%)	6%	2%	15%	4%	6%	10%	
C	235 (30%)	47%	25%	36%	25%	26%	30%	
**Rai stage (784 reported)**								**4.44E-06**
0	268 (34%)	26%	39%	22%	49%	44%	28%	
I	234 (29%)	18%	32%	34%	18%	28%	37%	
II	47 (6%)	8%	5%	9%	8%	2%	6%	
III	132 (17%)	34%	16%	17%	14%	17%	14%	
IV	103 (13%)	14%	9%	18%	11%	9%	15%	
**Lab tests (mean** + **SD)**								
Lymphocytes	38 (±54)	14 (±13)	28 (±27)	56 (±64)	24 (±38)	20 (±31)	54 (±66)	**1.74E-31**
Hemoglobin	13 (±1.8)	14 (±1.8)	14 (±1.4)	13 (±1.9)	14 (±1.5)	14 (±1.5)	13 (±2.0)	**1.67E-04**
Serum B2M	2.8 (±1.8)	2.3 (±1.2)	2.7 (±2.9)	3.4 (±2.1)	2.4 (±1.5)	2.4 (±1.1)	2.8 (±1.7)	**4.04E-11**
Serum LDH	480 (±240)	560 (±260)	500 (±180)	540 (±310)	380 (±140)	390 (±210)	530 (±180)	**8.78E-18**
**IGHV status (576 reported)**								**1.30E-05**
Unmutated	280 (49%)	35%	36%	68%	35%	44%	47%	
ZAP70 Positive (376 reported)	189 (50%)	35%	43%	60%	34%	60%	45%	**7.00E-03**
SF3B1 Mutated (211 reported)	34 (16%)	20%	10%	28%	15%	12%	13%	0.25
**Chromosome abnormalities (715 reported)**								
Deletion 11Q	100 (14%)	5%	11%	19%	11%	10%	17%	**0.03**
Deletion 13Q	273 (38%)	28%	45%	31%	47%	41%	40%	**0.05**
Trisomy 12	109 (15%)	25%	13%	18%	11%	22%	5%	**9.35E-05**
Deletion 17 P	68 (10%)	10%	11%	15%	6%	9%	5%	0.06
No major abnormalities	165 (23%)	35%	24%	19%	22%	17%	32%	**0.006**
**Immunophenotypic markers (mean** + **SD)**								
CD19	81 (±15)	68 (±19)	78 (±21)	87 (±10)	78 (±13)	77 (±14)	85 (±14)	**6.41E-17**
CD20	78 (±20)	84 (±21)	80 (±19)	77 (±21)	73 (±20)	78 (±21)	79 (±18)	**0.02**
CD22	63 (±39)	82 (±32)	70 (±41)	60 (±39)	68 (±36)	79 (±33)	43 (±39)	**1.14E-17**
CD23	87 (±18)	80 (±27)	89 (±18)	88 (±15)	86 (±22)	88 (±18)	86 (±15)	**9.95E-03**
CD38	24 (±27)	21 (±27)	36 (±39)	30 (±29)	17 (±22)	21 (±25)	21 (±25)	**0.01**
CD79b	43 (±38)	50 (±37)	43 (±34)	45 (±31)	39 (±30)	52 (±52)	31 (±28)	**2.68E-05**

The top part shows the distribution of diagnoses for the overall population (CLL and MSBL cases) and stratified by Signature group. Below that the presented information is based on only the CLL patients. Overall population and CLL Signature group descriptive statistics by diagnosis, age, race, gender, stage, cytogenetics, clinical lab tests, and outcomes. Signature group associations (*p*-values in right column) were determined by using chi-square and Kruskal–Wallis tests. Numbers represent the average and standard deviation. Percentages were calculated for data that was known. Significant statistical associations are highlighted with bold *p*-values.

The most notable difference between the groups relates to the distribution of the non-CLL diagnoses between SG-A and all the others (*p* = 3.66E-44). Group A is comprised of 52% MSBL diagnoses and 48% CLL cases whereas the remaining SGs are predominantly CLL (86–99%). There were significant differences in age, hemoglobin, platelets, % BM and PB lymphocytes and β2M (Supplementary Fig. S11) between the SG, but not for race (*p* = 0.84) or gender (*p* = 0.72) The SGs also associated with Binet (*p* = 0.00017) and Rai Staging (*p* = 4.4E-06), IGHV status (*p* = 1.3E-05), and chromosomal aberrations. Early stage cases (Binet A and Rai 0 and 1) are more prevalent in signatures E (68% Binet A), and F (60% Binet A), whereas SG-A has 47% Binet C. Overall 48% had unmutated IGHV, but unmutated cases were underrepresented in SG-A, -B and -D (35, 36, and 35%) and markedly overrepresented in SG-C (68%), ZAP70 positivity was seen in all SG, with overrepresentation in SG-C and underrepresentation in SG-D. In regard to mutations and cytogenetic aberrations, historically adverse del 11q and del 17p events (24% overall) were less common in SG-A, -B, -D, and E (15, 14, 16, and 17%) and overrepresented in SG-C (32%), while historically favorable 13q changes were seen across all groups as was Trisomy 12 (15% overall), although SGs A and E were enriched (25%, 22%) while SG-F was low (5%) for Trisomy 12. Flow cytometry measures showed differences for CD19, CD20, CD22, CD23, CD38, and CD79b.

### Signature groups clinical outcomes and associations

**Fig. 3:**
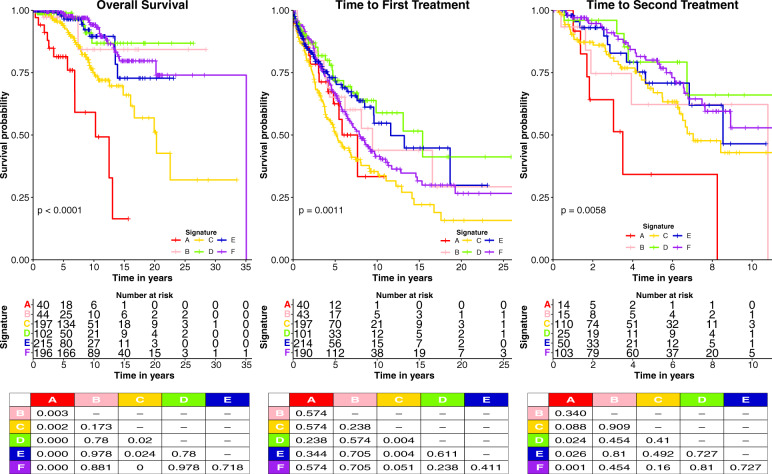
Outcomes of CLL signature groups. Kaplan–Meier plots of OS (left), TTFT (middle), and TTST (right) of CLL signature groups are shown. Overall log-rank *p*-values (bottom left of each figure), and BH corrected individual comparison *p*-values are shown (tables at the bottom). Signature groups A and C consistently have the lowest OS and TTFT compared to other groups.

The prognostic value of the SGs in CLL for OS, TTFT, and TTST were evaluated (Fig. 3). For OS, groups A and C had markedly inferior survival (*P* < 0.0001) (10.3 and 20.3 median years) relative to the other four groups, which were statistically similar to each other (Fig. 3A). First treatment occurred sooner for Groups A and C (5.8 and 5.23 median years) with group C being statistically earlier than all other groups, which were otherwise similar to each other. Additionally, the TTST was also inferior for Group A (median 3.5 years). Next, we assessed whether protein signature membership provided prognostic information beyond that of already identified prognostic markers. Multivariate analysis of signature groups and prognostic factors revealed that they are independently prognostic for survival outcomes (Table 2). When signature groups are included platelets, hemoglobin, chromosomal abnormalities, and IGHV status were no longer prognostic for survival outcomes. As shown in Supplementary Fig. S12, within each individual Rai stage there was heterogeneity based on SG, which was highly significant in Rai 0 and 1 (<0.0001, =0.0007 respectively) and the overall trend was similar in Rai 2 despite small numbers. Similarly, for TTFT, protein SG were prognostic within Rai stage 0 and 1. In contrast, within each individual SG, Rai staging was not prognostic (Supplementary Fig. S13). Therefore, Protein SG information was additive to Rai staging, but the converse was not true. We also performed a similar analysis for IGHV status, CLL-International Prognostic Index (IPI) classification, and mutational groups (i.e., Deletions 17p and 11q). We observed that IGHV status is prognostic within signature groups B and F for OS, all signatures except for E for TTFT (Supplementary Fig. S14). IPI classification did not prognosticate within any of the signatures for survival outcomes (Supplementary Fig. S15 top row), but did for TTFT in signature groups A, B, C, D, and F (Supplementary Fig. S15 bottom row). However, within a given IPI group signature group membership prognosticated for survival in all but the low-risk IPI group (Supplementary Fig. S16 top row), with small numbers hindering analysis of the very high-risk IPI group. Similar to the reverse analysis (Supplementary Fig. S15) the two scoring systems were complimentary for TTFT prognosis with protein signature group separating IPI low, high, and very high (Supplementary Fig. S16 bottom row). As for mutations, we observed that signature groups prognosticated within 17p deletion patients for OS and TTFT. Within those with 11q abnormalities, those with Sig C and F had worse survival than the other four signatures, with very small numbers of Sig A and B complicating the analysis (Supplementary Fig. S17). Proteomic SG membership was therefore additive to the prognostic information from IGHV-mutation status, 17p deletions, and CLL-IPI classification.

**Table 2:** Univariate and multivariate analysis of CLL prognostic factors for OS.

Variable	Category	OS Univariate (*n* = 794, number of events = 88)			OS Multivariate (*n* = 773, number of events = 85)		
		HR	95% CI	*p*	HR	95% CI	*p*
**Signature Group**	Signature A	**1**			1		
	Signature B	0.12	0.03–0.44	**1.26E-03**	0.09	0.02–0.44	**5.39E-04**
	Signature C	0.32	0.17–0.62	**6.96E-04**	0.27	0.13–0.57	**4.29E-05**
	Signature D	0.10	0.03–0.28	**1.47E-05**	0.13	0.04–0.39	**4.34E-05**
	Signature E	0.13	0.05–0.30	**3.23E-06**	0.12	0.05–0.31	**2.20E-06**
	Signature F	0.11	0.05–0.22	**2.63E-09**	0.11	0.05–0.25	**1.01E-08**
**Gender**	Female	1			1		
	Male	1.66	1.06–2.62	**0.03**	1.76	1.05–2.94	**0.01**
**IGHV**	Mutated	1			1		
	Unmutated	2.46	1.39–4.35	**0.002**	1.30	0.68–2.51	0.61
**Del 11Q**	Negative	1			1		
	Positive	1.85	1.07–3.21	**0.03**	2.63	0.93–7.43	0.60
**Chromosomal Abnormalities**	Any	1			1		
	None	0.50	0.28–0.88	**0.02**	0.97	0.32–2.97	0.96
**Trisomy 12**	Negative	1			1		
	Positive	2.24	1.32–3.82	**0.003**	2.58	1.01–6.55	0.71
**Del 13Q**	Negative	1.0					
	Positive	0.64	0.39–1.033	0.07			
**Del 17** **P**	Negative	1.0					
	Positive	1.99	1.14–3.48	**0.02**			
**Any 17**	Negative	1					
	Positive	2.48	1.00–6.16	0.05			
**Chr9**	Negative	1					
	Positive	2.04	0.64–6.46	0.23			
**SF3B1**	Positive	1					
	Negative	0.63	0.11–3.27	0.58			
**ZAP70**	Negative	1			1		
	Positive	2.81	1.61–4.93	**0.000296**	2.06	1.13–4.34	**0.02**
**Binet Stage**	A	1					
	B	1.14	0.64–2.30	0.68			
	C	1.60	0.96–2.56	0.05			
**Rai Stage**	0	1			1		
	I	1.17	0.67–2.05	0.57	0.88	0.47–1.66	0.70
	II	1.68	0.74–3.80	0.21	0.58	0.23–1.43	0.23
	III	1.38	0.65–2.93	0.40	0.31	0.12–0.86	**0.02**
	IV	2.22	1.15–4.26	**0.02**	0.82	0.36–1.88	0.63
**Age**		1.01	0.99–1.03	0.46			
**Lymphocytes**		1.00	0.99–1.00	0.69			
**B2M**		1.21	1.14–1.27	**3.34E-11**	1.19	1.09–1.31	**0.0001**
**Platelets**		1.00	0.992–0.999	**0.013**	1.00	0.99–1.00	0.45
**Hemoglobin**		0.85	0.77–0.94	**0.002**	0.89	0.77–1.03	0.12

Signature Groups, Chromosomal abnormalities, IGHV Status, Age, Gender, B2M, Lymphocyte counts, platelet counts, ZAP70 Status and Staging were evaluated. Hazard ratios (HR), 95% HR Confidence intervals, and *p*-values are displayed for each variable. The multivariate model included: signature groups, gender, IGHV status, Del 11Q, the presence of the four major chromosomal abnormalities in CLL, Del 17 P, ZAP70 status, Rai Stage, B2M, platelets, and hemoglobin levels.

### Signature groups can be leveraged to inform therapy decisions

**Fig. 4:**
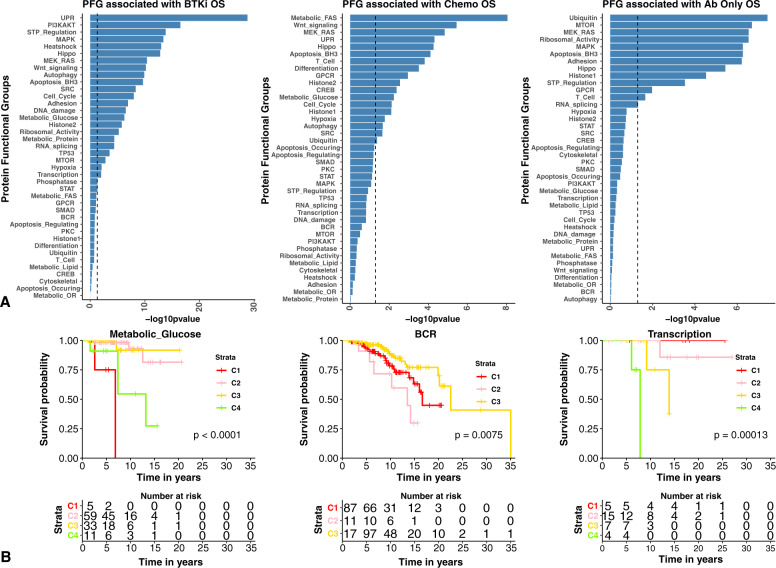
Protein functional groups associated with treatment response outcomes by therapy class. **A** Kaplan–Meier significance (*x*-axes) of protein functional group (*y*-axes) patient PFG clusters are shown in the bar plots. The dotted lines represent the -log10(0.05) *p*-value cutoff. As examples, the Kaplan–Meier plots of the Metabolic Glucose, BCR, and Transcription PFGs are also displayed for each therapy class (**B**).

**Fig. 5:**
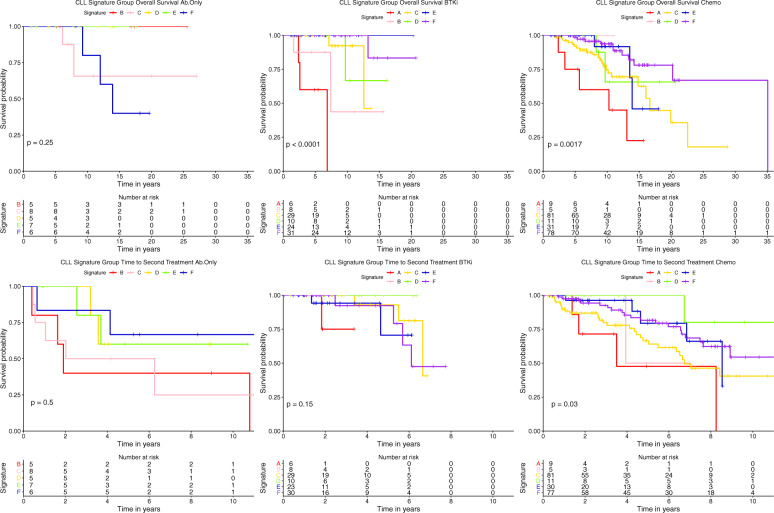
Signature group therapy responses. Signature Group OS (top panel) and TTST (bottom panel) were evaluated by therapy class (Antibody, Chemotherapy, and BTKi). For chemotherapy, signatures B-F would be good candidates whereas signatures A and C would not as they had shorter OS (top) and TTST (bottom). In regard to the BTKi class, most signature groups respond well (B-F). As the Antibody only group was small, it is difficult to make conclusions. Further studies are necessary for the signature groups responses to Ab therapy class.

As CLL therapy has shifted over time, former prognostic factors have become less relevant. As this data has a mixture of patients treated on older and more modern therapies we sought to determine if the proteomic classification remained informative independent of therapy. We therefore divided therapy into three broad classifications: BTK inhibition related protocols (±venetoclax), antibody only therapy, or chemo/chemoimmunotherapy regimens. As shown in Fig. 4, multiple PFG were prognostic (*P* ≤ 0.05) for OS within each therapy subclass; 25 with BTKi, 18 for chemo, and three for AB therapy. Interestingly Metabolic Glucose and BCR PFG clusters have survival disparities when treated with BTKi and chemoimmunotherapy. The Metabolic Glucose PFG has two favorable (C2-C3) and two unfavorable clusters (C1, C4) when treated with BTKi (Fig. 4 inner plots). Remarkably, the two unfavorable clusters have nearly opposite expression patterns of several glycolytic proteins (Supplementary Fig. S18) but share low expression of PRKAA1.2.pT172, implicating pathways activated by PRKAA1.2.pT172 in BTKi sensitivity or response. The SGs (Fig. 5) remain prognostic for OS regardless of therapy group, and for TTST for the chemo group. Thus, proteomic assignment is prognostic across therapy types at the level of PFG and SG. Of note, SG-A had the poorest OS in both chemotherapy and BTKi therapy types.

**Fig. 6:**
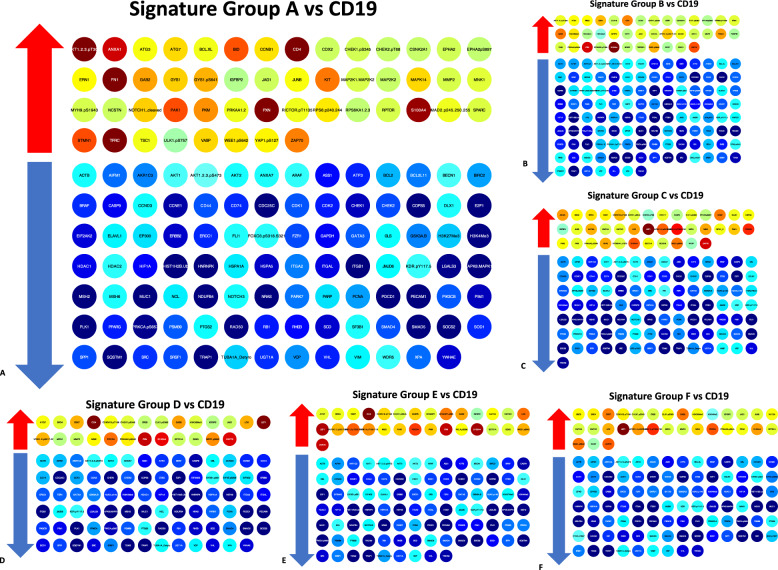
Potential therapeutic targets for CLL signature groups. **A**–**F** Proteins differentially expressed (*p* < 0.05) between CD19 controls and signature groups (designated by bold titles at the top of each panel). Upregulated proteins (adjacent to red arrows), that could serve as targets of inhibition, are represented with red–green–yellow colors based on median expression in each signature. Downregulated proteins (adjacent to blue arrows), which could serve as targets of replacement, are represented with cool blue colors.

Lastly, we endeavored to identify those proteins that were abnormally expressed within each SG with the hypothesis that this would identify potential therapy targets for inhibition or replacement. Differential expression of signatures versus the CD19 controls was performed using an ANOVA followed by Tukey HSD (Supplementary Table S4). The list of abnormally expressed proteins within each SG is shown in (Fig. 6) As suggested by Supplementary Fig. S5, CLL is characterized by more consistently downregulated proteins than universally upregulated. Many of these proteins have agents in development or clinical trial directed at them (Supplementary Table S5). This analysis provides a list of proteins that can be used for the development of targeted therapy strategies for each SG.

### Identification of a unique subset of CLL

A key finding is the identification of subgroups of CLL patients with a unique protein expression pattern (SG-A) that exhibits a distinctly inferior response to chemotherapy and BTKi (Fig. 4). The SG-A patients comprise 5% of all CLL patients. Also within this group are the majority of hairy cell leukemia (15/16) and half of the other small B-cell variant diseases (Fig. 3). Based on this we have called this Hairy Cell Proteomic Like CLL (HPLC). Notably, this group is not suggested by other traditional prognostic factors, as SG-A has low B2M, more normal hemoglobin and platelet counts, a lower percentage of adverse cytogenetics 17p and 11q alterations, and IGHV-nonmutation. SG-A is defined by constellations [1–3] that are minimally present in the other CLL, and by lower BCL2 and CD19 expression. Signature Group A CLL patients look like CLL histologically and immunophenotypically, but the association ends there as clinically they don’t respond well to any modality, while HCL is highly curable with the same. More insights, attempting to discern why they are so resistant will require future investigation.

### Identification of a classifier set of proteins

In order to be able to utilize this information clinically, the means to prospectively make a protein signature group assignment is required. We, therefore, wanted to identify a limited set of proteins that could be used to classify patients into signature groups. Using Random forest, we identified a set of 30 proteins that can distinguish between all signature groups (classification error rate of 18.6%) (Supplementary Fig. S19). Using our current model proteins, 77% of signature group A patients are classified as A, while 23% of signature group A patients are misclassified as other signatures (Supplementary Table S6). Misclassifying signature groups A and C patients into indolent groups (E and F) would be harmful, as those patients could not be treated immediately when necessary. However, there is minimal risk if signature groups D-F are misclassified as among each other. Upon further investigation of the misclassified patients, we found that misclassified SG-A had improved OS (median 12.5 years) compared to the accurately classified SG-A (median 6.9 years) and this was similarly observed in SG-D patients (Supplementary Figs. S20, S21). Additionally, we identified proteins driving outcome distinctions between correctly classified and misclassified patients (Supplementary Table S7) This misclassification serves to distinguish which SG-A and SG-D patients will do worse prognostically and the classifier set actually identifies an extremely poor prognosis cohort.

### PFG provide candidates for WaW diagnostics

**Fig. 7:**
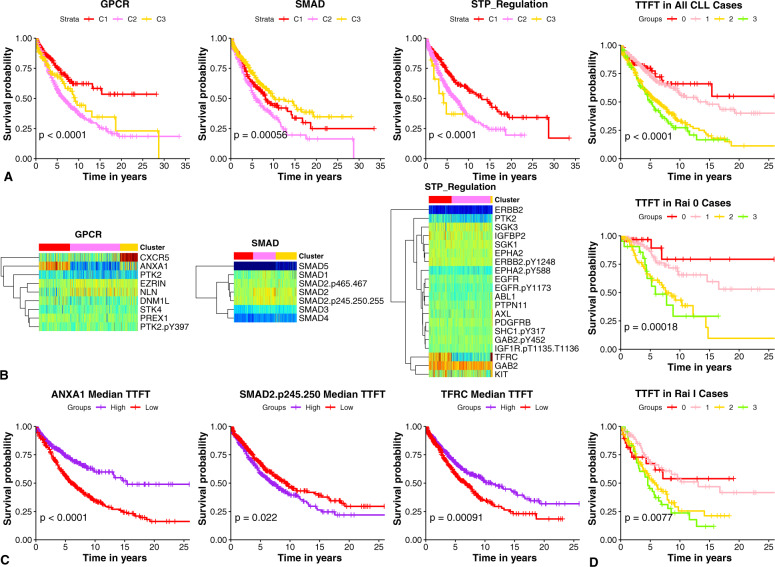
TTFT outcome disparities within GPCR, SMAD, and STP-regulation PFGs. Kaplan Meier plots of PFG clusters (**A**) and heatmaps of PFG protein members expression (**B**) TTFT Kaplan Meier Plots of ANXA1, TFRC, and SMAD2.p245 stratified by median (**C**). Low expression of ANXA1 and TFRC and high expression of SMAD2.p245 result in shorter TTFT. Kaplan Meier plots of the Accumulation of poor prognosis protein expression (ANXA1, SMAD2.p245, TFRC) (**D**). Groups represent the presence of 0–3 negative prognostic expression patterns (low ANXA1 or TFRC and high SMAD2.p245). The presence of two or more of these expression patterns results in shorter TTFT in all CLL (top), Rai stage 0 (middle), and Rai stage I (bottom) patients.

The indolent nature of some cases of CLL and lack of benefit from early intervention trials has made “Watch and Wait” (WaW) a standard approach for CLL patients that do not have indications for treatment initiation. However, with improved therapies, and better recognition of high-risk features, the appropriateness of WaW for select patients is less certain. We, therefore, asked if proteomic classification might identify subsets of patients that are more or less appropriate for a WaW strategy? We addressed this problem by evaluating PFG relationships with TTFT (Fig. 1A), observing that 18 of each were predictive for the respective outcome. For example, the GPCR, SMAD, and STP-regulation clusters were distinct in TTFT outcomes. In two of these PFGs (GPCR and STP regulation), shorter TTFT is being driven by low ANXA1 (Annexin1) and TFRC (transferrin receptor) expression, whereas high expression of SMAD2 and SMAD2.p245 drive similar outcomes within the SMAD PFG clusters (Fig. 7A–B). Since, ANXA1, TFRC, and SMAD2.p245 were prognostic individually (Fig. 7C), we tested whether they can be used in combinations to predict TTFT. Not only were they prognostic overall (Fig. 7D), but they were especially prognostic in early stage (Rai 0 and I) patients (Fig. 7D). Patients with ≤1 negative level of either of the proteins have a median TTFT of 14.59 years, whereas patients having 2–3 have a median of 5–6.27 years (*P*, 0.0001).

### Leukemia protein atlas

As a resource for others to interrogate and use we have posted all of the material from this study at the Leukemia Protein Atlas website: https://www.leukemiaatlas.org The dataset as well as figures for Protein functional group expression heatmaps, outcome Kaplan Meier curves, and Cytoscape networks from this study as well as from other RPPA based proteomic studies of leukemia can be found there.

## Discussion

Our main goal was to conduct proteomic profiling of CLL in a large cohort of patients to determine if we could establish a proteomic-based classification of CLL, and whether this would provide useful clinical information. Prior studies, using Mass spectrometry methodology in small cohorts (*N* = 14 and 16) suggested that proteomics could be informative in CLL, but lacked sufficient size for classification and prognostication [15, 19, 21]. Herein, we have presented data on the largest proteomic-based study of CLL with 795 unique CLL patients, using RPPA technology, probed for 384 antibodies. Importantly 21% of the antibodies were directed against PTM, enabling assessment of protein or pathway activation status, as well as total protein levels. Use of RPPA lacks the unbiased discovery potential of MS-based proteomics but allows for larger cohorts to be analyzed more rapidly. Protein expression in CLL was minimally affected by the time between diagnosis and sample collection, sample source, or whether the protein was prepared from fresh or cryopreserved cells. When normalized against normal CD19 + B-cells 16% of proteins had negligible expression across all patients, and while overexpression was common for many proteins, universal overexpression was uncommon (six proteins) At this gross level, we can conclude that proteins expression in CLL is markedly different from that of normal B-cells. There are many key findings from this study.

First, we confirm that we can classify CLL based on proteomics recognizing six signature groups, including a unique subgroup SG-A comprising 5% of cases, with resistance to all therapy modalities. Proteomic classification was largely independent of most demographic, clinical, and laboratory features typically assessed in CLL. It is not tightly correlated with Rai or Binet staging, but early stages were more common in SG-B, D, and advanced stages in SG-A and SG-C. The classification was also independent of laboratory measurements like serum β2M or LDH, or blood counts. There was more association with molecular events. Favorable IGHV-mutation status was significantly less common in SG-C (62% unmutated) and less common in SG-A, -B, and -D, and unfavorable Zap70 positivity higher in the prognostically adverse SG-C and E. The adverse cytogenetic finding of del 11q was uncommon in SG-A and del 17p was more common in SG-C, and while common cytogenetic events 13q and trisomy 12 were statistically significantly imbalanced between the groups, all events were seen across all groups. This observation that all these common molecular events occur in all groups may reflect that proteomics is assessing the net cumulative result of the sum of events in a given patient, and that this integrated signal, rather than the proteomic consequence of a single event is more relevant for classification. It is highly unlikely that this could be replicated by gene expression profiling from mRNA expression arrays or from RNA-seq as those assays cannot capture PTM, and as numerous studies in various cancers, (gastric, esophageal, AML, ALL) have shown, correlation is low (17–28%), therefore, proteomics is required to achieve this classification [22–25].

Second, we demonstrate that proteomic knowledge is highly prognostic in CLL, regardless of whether this is being evaluated at the level of individual proteins, related functional groups, or the system-wide signature, and is true for all three outcome measurements: OS, TTFT, or TTST. Among individual proteins, for OS, 15–18 times as many proteins were prognostic at a stringent *P* < 0.01 cutoff than random chance (*n* = 3.84) would predict, with similar findings for TTFT (~14x) and TTST (~3x). This greatly builds on findings from previous CLL studies as we identified several known (i.e., H3K27Me3, MCL1, and BCL2L11) and novel (i.e., NCSTN, SGK3, HSPD1, VTCN1, TRAP1, SOD1, and TAZ) proteins prognostic for OS, TTFT, and TTST [26–29]. Separate manuscripts for selected individual proteins are in process. These newly identified prognostic proteins suggest possible new potential therapeutic targets. The prognostic impact was greater when proteins were placed into functionally related groups and simultaneously evaluated. Among the PFGs, 78% were prognostic for OS, 45% for TTFT, and 48% for TTST, vastly exceeding chance at a *P* < 0.05 statistical cutoff. Analysis of individual PFGs reveals differential utilization of many therapeutically targetable protein groups, notably apoptosis and signal transduction pathway regulation and utilization. Finally, at the system-wide level, we identified six different signature groups, which again were prognostic for all three endpoints.

The prognostic impact of proteomics superseded that of other traditional prognostic features. Protein SG membership was still prognostic within each Rai stage group, but the converse was not true as Rai staging was not predictive within an individual SG. Within each SG IGHV-mutation status was not further predictive of OS but remained highly predictive for TTFT. SG membership also prognosticated for OS within all CLL-IPI classification groups except for low-risk patients, whereas IPI classification groups remained prognostic within signatures for TTFT. This demonstrates that the proteomic classification supersedes clinical-based staging and confirms the additive prognostic importance of protein signature classification to existing classifiers. In a multivariate model for OS, SG membership was independently predictive, while traditional prognostic factors, cytogenetic abnormalities, IGHV status, and stage were not. The protein SG also maintained prognostic significance when stratified by therapy class (antibody, chemo/chemoimmunotherapy, or BTKi), in contrast to many historical prognostic markers, which have lost significance with modern therapy.

Limitations of this study include a biased selection of proteins, small numbers of patients treated with modern modalities, and a lack of experimental validation of our findings. These weaknesses diminished our potential findings for therapy responses. The limitations can be overcome by repeating our analyses in a CLL/MSBL cohort, that is representative of disease population demographics and treated with modern therapy paradigms, using a broader number of target proteins.

Thus, in summary, proteomic classification provided new prognostic information, beyond that from traditional prognostic factors, relevant to both historical and modern therapy, for OS, TTFT, and TTST. The next critical step involves taking this information and translating it to the clinic. To do so first requires a means to rapidly classify patients at the time of diagnosis. Toward this end, we have developed a classifier based on the expression of 30 proteins that is 77% accurate for identifying SG-A and 87.5% for SG-C, the two most critical calls. We are working on developing a clinical kit for rapid clinical classification.

The second step needed for clinical utilization is selecting how to treat patients based on protein expression and SG membership. In general, it is easier to interfere with an upregulated protein than to replace the function of a protein that is under-expressed, so the six universally overexpressed proteins CHEK1.pS345, GAB2, IGFBP2, S100A4, WEE1.pS642, and ZAP70 are potential targets for investigation. CHEK1 and WEE1 are cell cycle proteins whose expression is commonly dysregulated in multiple cancer types [21, 30–32]. Phosphorylation of CHEK1 (serine residue 345) and WEE1 (serine residue 642) promotes the activation of DNA repair, cell cycle progression, and apoptosis. Previous studies have implicated the role of CHEK1 in CLL cell survival and proposed it as a potential therapy target for patients with 11q and TP53 mutations [33–36]. CHEK1 and WEE1 activation is required to initiate cell cycle arrest to give cancer cells enough time to recover from damage accrued from therapeutic agents [37]. As WEE1 is activated downstream of CHEK1, inhibition of either target would result in cumulative DNA damage and mitotic lethality. Since chemotherapeutic agents have cytotoxic effects, WEE1 and CHEK1 inhibitors could potentially be used in combination with them to increase efficacy. Due to the observed universal upregulation across all SGs, we propose CHEK1 not only be investigated as a target candidate for patients harboring 11q and 17p deletions but for all CLL patients. Prexasertib, a promising CHEK1 and CHEK2 inhibitor tested in ovarian cancer patients would be a candidate for treating CLL [38]. GAB2 is a GRB2-associated binding protein that normally promotes PI3K-AKT, MAPK, and MEK/ERK signaling in B and T cells [39]. Targeting GAB2 may serve as an alternative to mitigating PI3K/AKT signaling in addition to, or instead of, existing PI3K inhibitors Idelalisib and Duvelisib, perhaps with less problematic side effects. Both S100A4, a calcium-binding protein, and IGFBP2, an insulin-like growth factor-binding protein have roles in a multitude of cancer hallmarks including proliferation, angiogenesis, migration, invasion, and epithelial‑to‑mesenchymal transition, but their roles in CLL have not been characterized [40, 41]. This is the first study to propose IGFBP2, GAB2, and S100A4 as target candidates for CLL. As each has roles in promoting pathways essential to CLL pathogenesis, their inhibition would be problematic for CLL cell survival. ZAP70 is a tyrosine kinase with a role in T cell development and activation, proliferation, and migration [5, 42]. Targeting ZAP70 has been debatable as it is challenging to solely target the ZAP70 positive CLL cells to prevent the inactivation of anti-tumor T and NK cell responses [43].

Finally, we could also look to target proteins at the SG-level to facilitate this we generated lists of significantly over or under expressed proteins for each SG. This list can be cross-referenced with agents in development to identify overexpressed, or preferably significantly more activated (e.g., phosphorylated or other PTM) proteins, for each SG. This might suggest rational novel combinations to evaluate in selected subsets of patients. For example, in SG-A, AKT1.AKT2.AKT3.pT308, ANXA1, and FN1 are overexpressed and would make ideal target candidates.

CLL patients that do not require treatment are allocated to a WaW strategy. The proteomic classification identifies patients likely to have a shorter interval until treatment is required. Given the improvement in responses to modern therapy, it might be reasonable to offer to patients in those groups an early therapy intervention, when the disease is expected to have fewer resistance-associated mutations. When examining TTFT, we observed that disparities in the SMAD, GPCR, and STP-regulation clusters were driven by ANXA1 (low), TFRC (low), and SMAD2.p245 (high) expression. Notably, these proteins can be used in combination as a diagnostic tool to identify patients who will need to be treated sooner. Patients with abnormal expression of 2 or more of these proteins had a median TTFT of 5 years whereas patients with <2 had a median of 14.5 years. Therefore, we propose TFRC, ANXA1, and SMAD.p245 as biomarker candidates for WaW determinations.

The final major observation was that there is a small group, SG-A, comprising 5% of all CLL cases with a different proteins expression signature that appears like that of HCL. Outcomes for patients in this group for CLL as well as for PLL and MCL patients were worse than patients in the other SG. This suggests that proteomics is needed to identify this highly adverse group [44]. There is a paradox as HCL is nearly uniformly responsive to the purine analog cladribine or anti CD20 antibody rituximab, yet these CLL, PLL, and MCL patients with the same proteomic profile are not. The HPLC patients also do poorly with BTKi therapy, suggesting the need to identify these patients and triage them to novel therapies.

Finally, this report only covers a tiny portion of the information that was generated. We have placed all of this data online at our Leukemia Protein Atlas website for all to explore as a public resource. We look forward to assisting others with analyzing this data.

## Methods

### Patient population

Frozen (*n* = 744) and fresh (*n* = 127) blood (*n* = 799) and bone marrow (*n* = 71) samples were acquired from patients diagnosed with CLL(*n* = 795), HCL(*n* = 12), HCLV(*n* = 4), LGL-T(*n* = 4), MBL(*n* = 4), MCL(*n* = 12), MZL(*n* = 12), PLL(*n* = 4), Richter’s (*n* = 8), T-cell PLL(*n* = 16) at MD Anderson Cancer Center (MDACC) between 2005 and 2019. The samples were collected under protocols Lab01-473, LAB03-0893, Lab 04-0678, Lab08-0431 and Lab07-0719 in accordance with protocols approved by the Institutional Review Board (IRB) of M.D. Anderson Cancer Center. Informed consent was obtained in accordance with the Declaration of Helsinki. Samples were collected within a year (*n* = 435), 5 years (*n* = 286), 10 years (*n* = 91), after 10 years (*n* = 52), and >20 years (*n* = 7) after initial diagnosis. These incudes patients who were never treated (*n* = 527) or treated within 100 days (*n* = 45), a year (*n* = 45), 1–2 years (*n* = 43), 2–5 years (*n* = 115), and >5 years (*n* = 93) after diagnosis. As the Richter’s cases are intermixed with the CLL cases, this suggests that the proteomics of the circulating CLL cells, in a patient that has developed Richter’s transformation, have not been markedly changed.

### RPPA methodology

RPPA was used to create proteomics profiles of 871 patient samples (as described above) and five CD19 + controls (derived from five healthy donors). Proteins were isolated from PBMCs in frozen (*n* = 744) and fresh patient samples (*n* = 127). Fresh samples were processed by being layered on Ficoll and centrifuged to remove neutrophils and red blood cells, washed in PBS, centrifuged, and counted. Frozen samples were initially processed in the same manner except they were later thawed, placed in fresh media, layered on Ficoll and centrifuged to remove dead cells, washed with PBS and centrifuged and counted. The cells were lysed to produce whole cell lysates as previously described and normalized to a concentration of 1 × 10^4^ cells/μL [45]. CLL sample lymphocyte purity (median, 97%) after Ficoll separation was estimated using lymphocyte counts (Supplemental Fig. S22). Five serial two-fold dilutions (1:1, 1:2, 1:4, 1:8, 1:16) of each patient, cell line, or control sample was printed onto slides. The slides were probed with 384 strictly validated primary antibodies and a secondary antibody conjugated to an infrared molecule to amplify and detect the signal. Antibodies that had been previously validated (*n* = 377) were selected based on potential relevance to CLL, other leukemias, or other cancers and five newly validated antibodies relevant to CLL (i.e., BTK, ZAP70, SF3B1, phospho-SF3B1, phospho-BCL2.pSer15) were also validated using previously described methods [46]. The 384 antibodies targeted: 302 total protein, 72 phospho-proteins, four cleaved proteins (CASP3, CASP7, NOTCH1, and PARP), and six targeting Histone 3′ methylation sites. A “Rosetta Stone” list of all antibodies used, the catalog number, manufacturer’s name, HUPO name, and the concentrations of primary and secondary antibodies used are shown in Supplementary Table S8. Stained slides were quantitated using Microvigene software (Version 3.4, Vigene Tech).

### Data processing, normalization, and quality control

The SuperCurve R package was utilized to calculate a single value of protein concentration from the five serial dilutions on a log 2 scale [47]. The quality of the staining procedure was further assessed by examining the Supercurve images and identifying and eliminating slides without sufficient variation in signal or which lacked the expected sigmoidal curve. Loading control and topographical normalization procedures were performed to account for protein concentration and background staining variations. The data were normalized by subtracting the median of the rows and columns across all samples to ensure that sample protein expression estimated from different slides can be compared [48]. Lastly, the median of CD19 control proteins was subtracted to normalize values to a normal median of zero enabling recognition of whether expression in the patient was within, above or below that of normal.

### Computational analysis

The data was considered at three different levels: as individual proteins, within functionally related groups, and collectively to generate a “systems biology” approach utilizing the Metagalaxy method similar to previous studies [45, 49–51]. The 384 antibodies were sorted into 40 protein functional groups (PFG), based on functions reported in the literature or based on expression correlation with other proteins. The progenyClust R package (version 1.2), a bootstrapping and stability-based method, and k-means clustering were used to identify the optimum number of unique protein functional group expression patterns in the patient population. Linear discriminant (LDA) and principal component analyses (PCA) were used to compare the patient clusters to the normal CD19 B control samples.

To build the Metagalaxy, encompassing all the protein data at the PFG level, a matrix was created by assigning patients binary classifications (1 for present, 0 for absent) based on their protein functional group expression patterns. Block clustering (version 4.4.3) was used on the data matrix to identify repetitive co-occurring PFG expression patterns (constellations) and groups of patients with a similar combination of constellations to recognize signatures. The optimal number of protein constellations and signatures were obtained by calculating the largest sum of the squared difference between the expected and observed values, divided by the expected value of each box (coordinate between a signature and a constellation). The expected value was defined as the sum of cluster membership within a constellation, divided by the proportion of patients in a given signature. Patient signatures were further classified into “signature groups” based on similarities in their constellations and clinical outcomes.

At each analysis level, individual protein, PFG, Metagalaxy, relationships between categorical, and numeric clinical characteristics were evaluated using Chi-square, Fisher exact, and the Kruskal–Wallis tests. Prior to applying non-parametric statistics, we determined that our numeric clinical traits, being compared across the signature groups, were not normally distributed nor had equal variance using the Shapiro–Wilk and Levene tests. For outcome analysis Kaplan–Meier was used to assessing signature group and PFG cluster relationships with overall survival (OS), time to first treatment (TTFT), and time to second treatment (TTST). *P*-values from multiple testing of proteins and PFGs were false discovery rate (FDR) corrected. To evaluate whether signature groups prognosticate within CLL-IPI risk groups and vice versa, we first used the CLL-IPI point system criterion to categorize CLL patients into low (0–1), intermediate [2, 3], high [4–6], and very high [7–10] risk groups and used a Kaplan Meier test [52]. Patients with missing age, stage, IGHV status, β2 M, or 17p deletion information were excluded from the CLL-IPI analysis. A similar analysis was performed for signature groups using Rai stage, IGHV status, and mutational groups (11q and 17p). Prognostication of individual proteins was tested and validated by iterative testing (*n* = 200) of optimum cox hazard models (median, tertile, sextile, or continuous) on randomized training (66%) and test (33%) sets of CLL RPPA data. Random forest was used to bin patients into expression groups (median, tertiles, sextiles) based on the training data. The optimum model was determined based on Harell’s c-index. Differential expression between the signature groups and CD19 controls was performed using a one-way ANOVA and Tukey honest significant difference test. Differentially expressed proteins were visualized in Cytoscape. Random forest was used to select proteins that can be used to classify patients into signature groups (randomForest v 4.6–14). All the statistical tests and plots were generated in R (Version 3.6.1).

## Supplementary information

Supplemental Figures and Legends

Supplementary Table 1 - Table of Individual Protein Cox hazard Results

Supplementary Table 2 - Table of Protein Functional Groups and Protein Members

Supplementary Table 3 - Table of Constellations and their Protein Functional Group Expression pattern membership

Supplementary Table 4 - Table of Differential Expression Results for Signatures and CD19 controls

Supplementary Table 5 - Table of FDA approved drug targets for overexpressed proteins in Signature Groups.

Supplementary Table 6 - Random Forest Model Signature Group Classifications

Supplementary Table 7 - Differential Expression of Signature Group Correctly Classified and Misclassified patients

Supplementary Table 8 - Rosetta Table of antibodies used for the RPPA

## Data Availability

Code for progenyclust and Metagalaxy are available on the Leukemia atlas website: https://www.leukemiaatlas.org/code.
