# Supplementary material for: Proteomic profiling based classification of CLL provides prognostication for modern therapy and identifies novel therapeutic targets
Source: Blood Cancer J. 2022 Mar 17;12(3):43. doi: 10.1038/s41408-022-00623-7 (PMC8931092; doi:10.1038/s41408-022-00623-7)

Supplemental Table Legends

Supplementary Table S1: Table of Individual Protein Cox hazard Results(.xlsx). Hazard ratios (hr), 95% confidence intervals (CI), p-values (p-value), and FDR p-values are provided for each clinical outcome (OS, TTFT, TTST, and BTKi OS)

Supplementary Table S2: Table of Protein Functional Groups and Protein Members (.docx)

Supplementary Table S3: Table of Constellations and their Protein Functional Group Expression pattern membership (.docx)

Supplementary Table S4: Table of Differential Expression Results for Signatures and CD19 controls (.xlsx). Mean differences and Tukey pairwise comparison adjusted p-values are listed for 384 proteins in all comparisons. Zero p-values represent p-values that were less than 2.2x10e-16.

Supplementary Table S5: **Table of FDA approved drug targets for overexpressed proteins in Signature Groups(.xlsx)**. A combined list of proteins, that were upregulated in each signature group, was used as input into the Drug Gene Interaction Database (DGIdb, https://www.dgidb.org). Protein name, drug, and interaction types are shown.

Supplementary Table S6: **Random Forest Model Signature Group Classifications(.xlsx)**. Columns and rows (left) represent the number of signature group patients that were classified

based on the model proteins. The number of patients and prediction error rates are in the columns on the right.

Supplementary Table S7: Differential Expression of Signature Group Correctly Classified and Misclassified patients. (.xlsx) Protein names, log fold change (LFC), Wilcoxon test p-values, and FDR corrected p-values are provided for each comparison.

Supplementary Table S8: Rosetta Table of antibodies used for the RPPA(.xlsx). Protein names, manufacturing company, product or catalog numbers, primary, and secondary antibody dilutions are reported.

Supplemental Figure Legends (all PDF images)

Supplementary Fig S1: **Principal Component Analyses of RPPA Samples and Possible Confounders**. A-D) PCA of RPPA samples by diagnosis, collection intervals (within 1,5,10, and 20 years), TX status, and condition and organ. E) PCA of CLL patients with matched blood and bone marrow samples taken on the same day. The grey lines connect paired samples from the same patient. A follow-up t-test assessing all proteins confirmed that there were minimal differences between matched CLL blood and bone marrow samples (0 significant proteins). F) Confounder Variance Estimation. The variancePartition R package was used to estimate the percentage of variance (via linear models) contributed by each variable across all proteins. CLL sample condition (i.e., fresh, frozen), collection interval (i.e., within 1,5,10, and 20 years), age, TX status, Rai stage, organ (i.e., blood and bone marrow), and gender contribute minimally to the variation observed across all proteins (median range 0-2%). The majority (median, 89%) of the residual protein expression variance is biological.

Supplementary Fig S2. **Heatmap of all proteins in the CLL RPPA dataset.** CD19 normalized proteins were Ward linkage clustered and stratified by signature group and diagnosis (annotations at the top). The blue-green-yellow-red heatmap color scale represents protein expression that is below (blue), average, or above (yellow, orange, red) normal CD19 protein expression.

Supplementary Fig S3. Proteins Associated with Outcomes. Proteins significant ($p<0.01$) for OS, TTFT, and TTST by median (left) and tertile (right) are listed. Highlighted proteins are consistently significant after FDR correction ($p<0.05$).

Supplementary Fig S4: Outcome based on SOD1 expression levels. Overall Survival and Time to First Treatment outcomes (in years) based on SOD1 expression separated by median (panel B) and tertile (panel A) expression levels (denoted in figure legends on the right). By tertile and median, higher expression of SOD1 negatively affects TTFT and OS.

Supplementary Fig S5. Proteins Associated with BTKi Overall Survival. Proteins significant ($p<0.05$) for OS by median (left), tertile (middle), septile (right) are listed. Blue highlighted proteins are consistently significant after FDR correction.

Supplementary Fig S6. Protein Functional Group Expression Patterns present in CLL and CD19+ controls. The optimum number of clusters for each PFG (second column), normalized against normal CD19+ B cells are displayed. Each PFG cluster is further characterized by whether

this pattern was similar to a pattern seen in the normal control (checked patterns) or was unique to CLL (solid). Compared to CD19 controls, all Ubiquitin and PKC patterns are leukemia specific.

Supplementary Fig S7: Protein Function Groups with One Odd Cluster. Kaplan Meier overall survival curves of 16 PFGs with one cluster that stands out from the rest.

Supplementary Fig S8: Protein Function Groups with Heterogeneous Outcomes. Kaplan Meier overall survival curves of 15 PFGs without differences in survival outcomes.

Supplementary Fig S9: Protein Function Groups that were not Prognostic. Kaplan Meier overall survival curves of 9 PFGs without differences in survival outcomes.

Supplementary Fig S10: Overall Survival of Numeric CLL Signatures (1-16). OS information along with constellation similarities was used to merge patient signatures into six signature groups (A-F). Signatures 1 and 2 were merged to form SG-A. Signatures 3 and 4 formed SG-B. Signatures 5-7 formed signature group C. SG-D consists of signatures 8-11. SG-E consists of signatures 12-13, and lastly SG-F consists of signatures 14-16.

Supplementary Fig S11: CLL Signature Group Lab Test Boxplots. Boxplots of Lymphocyte Counts, Hemoglobin, B2M, and LDH. Kruskal-Wallis p-values are at the top left.

Supplementary Fig S12: **Signature Group outcomes stratified by Rai stage**. Signature Group OS (top panel) and TTFT (bottom panel) separated by Rai stages (0-IV). Signature groups are prognostic within each Rai stage

Supplementary Fig S13: Overall survival of Rai stages stratified by signature group. Rai staging is not a prognostic indicator within any of the signature groups.

Supplementary Fig S14: OS, TTFT, and TTST of IGHV Status Within Signature Groups. Log-rank Kaplan Meier analysis was performed on patients stratified by IGHV status within signature group (A-F) for OS (first row panel), TTFT (second row panel), and TTST (third row panel).

Supplementary Fig S15: CLL-IPI Classification Group outcomes stratified by Signature Group. Kaplan Meier plots of CLL-IPI risk group OS (top panel) and TTFT (bottom panel) outcomes within each signature group.

Supplementary Fig S16: Signature Group outcomes stratified by CLL-IPI Classification Group. Signature Group OS (top panel) and TTFT (bottom panel) separated by CLL-IPI risk groups (Intermediate, Low, High, and Very High). Signature groups are prognostic within each Rai stage.

Supplementary Fig S17: Signature Group outcomes stratified by 17p and 11q deletion grouping. Signature group OS (left) and TTFT (right) outcomes were evaluated within patients who had 17p(top) and 11q (bottom) deletions.

Supplementary Fig S18. Heatmap of Metabolic Glucose Protein Functional Group Members. Four patient clusters (C1-C4) are represented based on the expression patterns of member proteins (annotations at the top). Proteins are clustered based on ward linkage (left). The color scale represents whether proteins are expressed above (warm colors) or below (cool colors) normal levels.

Supplementary Fig S19: Heatmap and Dot plots of Discriminative Proteins. Heatmap of protein median expression (panel A) and dot plot of Mean Decrease accuracy scores of proteins that can distinguish between signature groups (panel B).

Supplementary Fig S20: **Overall Survival of classified and misclassified SG-A patients**. The 9 misclassified patients had fewer deaths (2 vs 10) and improved OS (median 12.5 versus 6.9 years) compared to correctly classified SG-A patients

Supplemental Fig S21: Overall Survival of Misclassified Signature Group Members

To investigate why signature group patients were misclassified by our random forest model, log-rank Kaplan Meier analyses were performed to test whether there were differences in survival outcomes. We particularly looked for outcome differences between patients from good prognostic groups (Signatures BDEF) who were classified into bad groups (Signatures A-C) and vice versa.

Misclassified SG-A patients (panel A, blue) had better overall survival whereas misclassified SG-D (panel D, blue) patients did worse. Misclassified patients in SGs B, C, E, and F (panels B, C, E, F) did not display differences in survival outcome

Supplemental Fig S22. RPPA Sample Purity Boxplot. All sample (blue) and CLL (red) sample lymphocyte purity are displayed for all (left, n=871) and CLL patient samples (right, n= 795). All samples refer to MSBL and CLL cases together. The x-axis displays the groups and y-axis displays percentage. Lymphocyte sample purity was estimated by dividing the percentage of peripheral blood (PB) lymphocytes (B and T cells) by the percentage of PB monocytes + lymphocytes. The median lymphocyte sample purity across all (97.40%) samples and CLL (97.47%) were similar. Therefore, most of the detected protein signal in the CLL samples stem from the B cells.

Supplemental Figures

Supplemental Figure S1: Principal Component Analyses of RPPA Samples and Possible Confounders

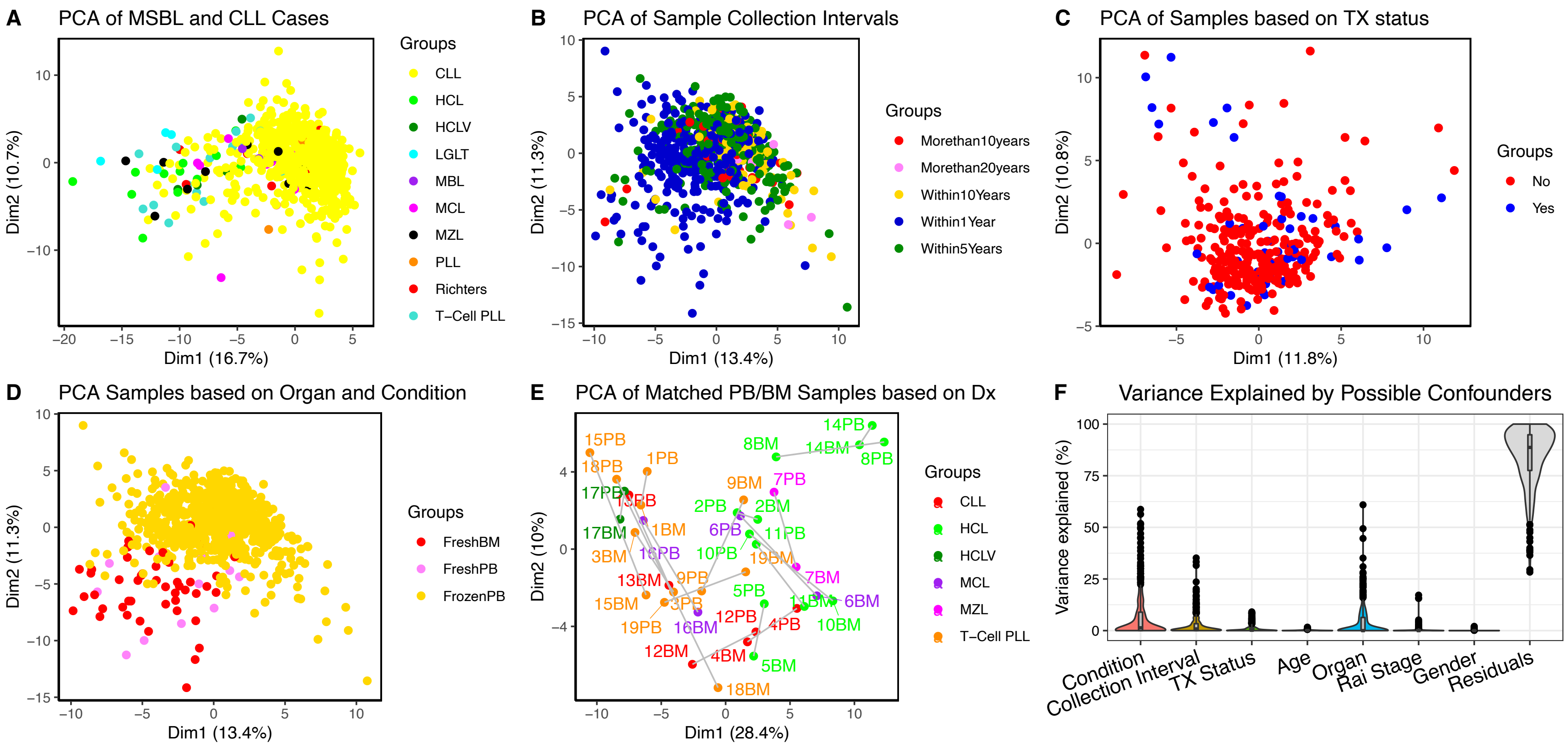

Supplementary Fig S2. Heatmap of all proteins in the CLL RPPA dataset

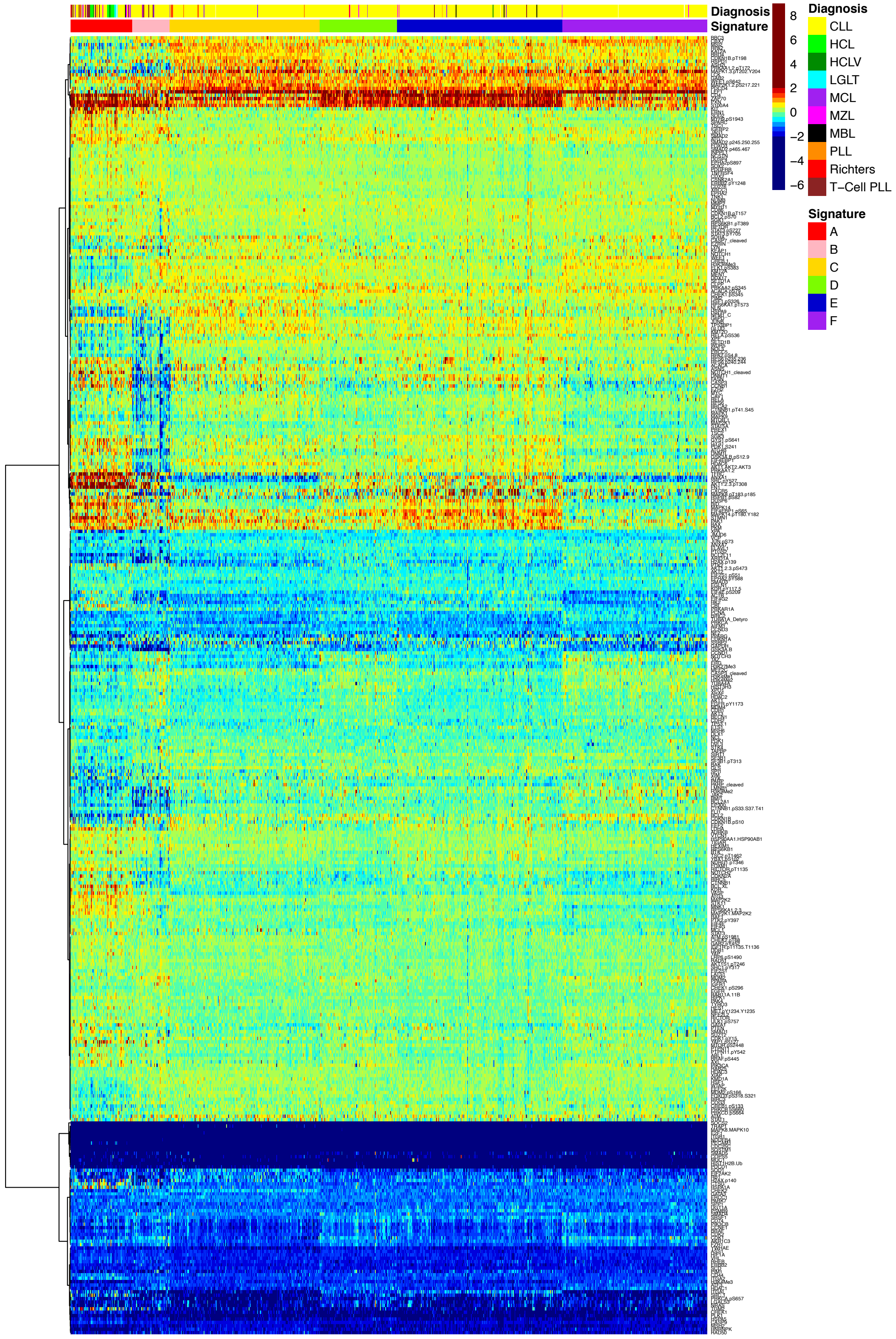

Supplementary Fig S3 Proteins independently predictive of outcome (FDR p<0.05)

Median					
Overall Survival (N=59)		Time to First Treatment (n=52)		Time to Second Treatment (N=11)	
Protein	pvalue	Protein	pvalue	Protein	pvalue
AIFM1	0.008	ACACA.pS79	2.24E-03	AKT1S1.pT246	1.79E-04
AKT2	0.004	AIFM1	2.47E-06	ARAF	8.13E-03
ARAF	7.52E-04	AKT1	4.31E-04	ARID1A	4.99E-04
ARID1A	1.11E-04	AKT1.2.3.pT308	2.53E-06	DNM1L	2.13E-03
ATG4B	4.15E-04	AKT1.AKT2.AKT3	4.66E-03	FOXO3	3.23E-04
BBC3	6.26E-03	ANXA1	6.90E-08	GATA1	9.64E-03
BCL2L1	2.00E-03	ATM.pS1981	3.30E-04	H3K27Me3	3.34E-04
BCL2A1	5.73E-03	BAX	2.46E-03	MDM4	7.83E-03
BCL2L11	3.14E-03	CASP3	2.04E-03	NFE2L2	6.44E-04
CASP3	3.46E-08	CASP9	6.52E-03	PARK7	7.83E-03
CASP9	7.31E-05	CCNE1	4.47E-03	VTCN1	7.36E-03
CBX7	3.20E-04	CD4	2.13E-04		
CCNB1	6.23E-03	CD74	6.53E-03		
CD74	4.89E-03	COG3	4.11E-03		
CDK1	2.50E-03	DDB1	3.07E-07		
CDX2	5.46E-03	DNMT1	5.47E-03		
CHEK2.pT68	7.50E-03	EIF2AK2	6.65E-04		
CREB.1	1.25E-03	EIF2S1.pS51	5.20E-03		
CSNK2A1	6.58E-03	ERN1	5.84E-06		
DNM1L	2.68E-06	EZRIN	2.34E-03		
DUSP4	1.52E-04	GLS	1.74E-03		
EIF2AK2	9.99E-03	H2AX.p139	1.68E-03		
EIF4E	1.40E-04	H2AX.p140	5.39E-04		
EIF4G	8.35E-04	H3K27Me3	3.79E-04		
EZH2	8.62E-04	H3K4Me1	1.21E-03		
FLI1	1.05E-03	HIST3H3	4.50E-03		
GYS1	9.85E-03	HK2	4.76E-03		
H3K27Me3	1.74E-05	HSPD1	5.37E-03		
H3K36Me3	1.55E-04	ITGAL	1.16E-04		
HES1	3.83E-03	KDR	8.29E-03		
HSPD1	2.35E-04	LGALS3	1.08E-04		
JAK2	1.17E-04	MAPK14	2.86E-03		
LATS1	6.96E-03	MAPK14.pT180.Y182	2.91E-03		
LDHA	3.45E-03	MSI2	3.85E-04		
LRP6.pS1490	3.63E-03	NLN	1.11E-03		
MAP2K2	5.60E-03	PAK1	4.27E-03		
MCL1	1.80E-04	PDCD4	4.71E-03		
MDM4	3.64E-03	PECAM1	5.99E-03		
MEF2C	6.03E-04	PIM2	1.92E-04		
MNK1	9.65E-06	PPARA	5.21E-04		
MUC1	1.50E-04	PPARG	4.72E-03		
NOTCH3	2.01E-03	PTEN	5.23E-03		
PAK1	3.83E-04	PTK2	6.50E-03		
PCNA	1.51E-04	RPS6KB1.pT389	6.15E-04		
PIM1	7.67E-03	SGK3	9.01E-05		
PIM2	1.55E-04	SOD1	1.86E-04		
PKM	0.007	SPARC	2.01E-03		
PTGS2	0.003	SRC.1	8.13E-05		
RPS6KB1.pT389	4.51E-05	SRC.pY527	1.23E-03		
SGK3	0.001	STAT1	3.15E-03		
SOD1	6.17E-05	TFRC	9.07E-04		
SPI1	0.007	ZAP70	7.10E-07		
SRSF1	0.001				
STAT1	0.002				
STK11	0.006				
TUBA4A	0.004				
VASP	0.008				
VTCN1	0.004				
ZAP70	1.14E-06				

Tertile					
Overall Survival (N=78)		Time to First Treatment (N=56)		Time to Second Treatment (N=14)	
Protein	pvalue	Protein	pvalue	Protein	pvalue
AIFM1	1.61E-03	AIFM1	5.86E-05	ARAF	6.87E-03
AKT1	9.42E-03	AKT1.2.3.pT308	1.94E-06	EIF4G	4.24E-03
AKT1.2.3.pT308	9.35E-03	AKT2	1.22E-03	FOXO3	3.58E-03
ARAF	1.10E-03	ANXA1	4.48E-07	GAB2.pY452	3.27E-03
ASNS	1.04E-03	ATM.pS1981	1.61E-03	H2AX.p139	5.53E-03
ATG4B	5.10E-03	BAX	1.67E-03	H3K27Me3	5.41E-04
BAX	7.23E-03	BCL2A1	6.17E-03	H3K9Me2	3.29E-03
BBC3	5.54E-03	BCL2L11	4.78E-03	NCSTN	8.51E-04
BCL2L1	8.82E-04	CASP3	1.35E-04	NFE2L2	1.13E-03
BCL2	4.36E-03	CCNE1	3.26E-03	PLK1	6.96E-03
BCL2L11	1.67E-04	CD4	1.06E-05	STAT3.pS727	5.12E-03
BID	5.44E-03	CD74	1.86E-03	TAZ	4.72E-04
BIRC2	1.50E-03	CHEK1.pS296	2.28E-03	TRAP1	1.71E-03
CASP3	1.96E-05	COG3	8.31E-03	VTCN1	6.26E-03
CASP3_cleaved	4.89E-03	DDB1	2.72E-06		
CASP9	4.43E-04	DDX17	9.47E-04		
CBL	2.42E-03	ERN1	1.37E-04		
CBX7	5.21E-04	EZRIN	2.11E-04		
CCNB1	2.69E-04	FN1	1.48E-04		
CD74	7.69E-03	GAB2	4.56E-05		
CDKN1B	9.92E-03	H2AX.p139	6.71E-03		
CHEK1	5.79E-04	H2AX.p140	9.29E-04		
DDB1	4.09E-03	H3K27Me3	2.72E-03		
DNM1L	8.74E-05	H3K4Me1	5.71E-03		
DNMT1	1.32E-03	HNRNPK	5.02E-03		
DUSP4	3.94E-03	ITGAL	5.06E-04		
EGFR.pY1173	9.19E-03	KAT2A	1.69E-03		
EIF2AK2	3.83E-03	LGALS3	2.28E-04		
EIF2S1.pS51	8.27E-03	MAPK14	1.53E-04		
EIF4E	3.85E-03	MAPK14.pT180.Y182	2.01E-04		
EIF4G	7.09E-04	MSI2	2.60E-04		
EPHA2.pY588	3.69E-03	NLN	1.12E-04		
EZH2	1.39E-06	PAK1	1.32E-03		
FASN	5.54E-03	PARP	6.27E-03		
FLI1	4.23E-04	PARP_cleaved	2.75E-04		
H2AX.p140	3.41E-04	PECAM1	6.13E-04		
H3K27Me3	4.28E-06	PIM1	2.86E-03		
H3K36Me3	3.83E-05	PIM2	1.63E-04		
H3K4Me1	2.67E-03	PPARA	6.21E-03		
HDAC2	3.22E-03	PTEN	8.81E-03		
HSPD1	1.45E-03	PTK2	7.27E-03		
JAK2	2.41E-03	RAD50	5.98E-04		
LDHA	4.20E-04	RB1	2.01E-03		
MAP2K1.2.pS217.221	9.03E-03	RPS6	9.21E-03		
MAP2K2	6.64E-04	RPS6KB1.pT389	6.12E-04		
MCL1	4.97E-05	SDHA	1.75E-03		
MDM2.pS166	8.95E-03	SGK3	1.50E-05		
MEF2C	2.09E-04	SMAD5	2.71E-04		
MNK1	4.92E-08	SOD1	1.78E-04		
MUC1	1.13E-04	SPI1	8.02E-03		
NCSTN	4.10E-03	SRC.1	4.03E-07		
NOTCH3	4.12E-03	SRC.pY527	5.55E-04		
NUMB	8.41E-03	TFRC	4.83E-06		
PAK1	3.28E-03	TRAP1	2.88E-03		
PCNA	2.75E-03	TSC2	8.48E-03		
PDGFRB	5.73E-05	ZAP70	2.13E-09		
PIM1	9.15E-03				
PIM2	3.29E-05				
PKM	1.08E-03				
PPARA	7.30E-03				
PTGS2	3.10E-03				
RPS6KB1.pT389	6.06E-03				
SF3B1	8.01E-05				
SGK3	2.61E-04				
SMAD2	8.09E-04				
SMAD2.p245.250.255	4.64E-03				
SOD1	2.25E-03				
SPI1	9.92E-05				
SPP1	5.53E-03				
SRSF1	2.47E-04				
SSBP2	4.94E-03				
STAT1	1.38E-03				
STK11	2.50E-03				
TAZ	7.58E-03				
TRAP1	8.80E-05				
TUBA1A_Detyro	6.16E-03				
TUBA4A	1.04E-03				
ZAP70	1.67E-05				

Sextile					
Overall Survival (N=79)		Time to First Treatment (N=45)		Time to Second Treatment (N=18)	
Protein	pvalue	Protein	pvalue	Protein	pvalue
ACACA.pS79	7.83E-03	AIFM1	2.00E-06	AKT1S1.pT246	4.60E-03
AIFM1	6.26E-03	AKT1.2.3.pT308	3.28E-05	ARID1A	5.04E-03
AKT1.2.3.pT308	1.05E-06	ANXA1	2.51E-06	BCL2A1	4.22E-03
ARAF	4.05E-04	ASNS	7.17E-03	CBX7	1.70E-04
ARID1A	6.88E-03	BAX	4.94E-03	CSNK2A1	3.86E-03
ASNS	1.11E-03	BBC3	1.80E-03	DNM1L	8.52E-03
ATG4B	3.85E-03	BECN1	5.82E-03	DUSP4	8.30E-03
BAK	1.14E-03	CASP3	1.21E-03	H2AX.p139	3.76E-03
BCL2L1	2.93E-03	CCNE1	3.91E-03	H3K27Me3	3.71E-05
BCL2L11	9.27E-05	CD4	5.19E-06	H3K9Me2	2.22E-04
BIRC2	7.60E-05	DDB1	1.86E-05	MAP2K2	3.01E-03
CASP3	8.55E-06	EEF2K	1.98E-03	MAPK14	6.99E-03
CASP9	2.19E-03	ERBB2	9.99E-03	NFE2L2	1.07E-03
CBL	2.67E-03	ERCC1	8.48E-03	PLK1	6.17E-03
CBX7	1.11E-04	ERN1	1.11E-04	SMAD2	1.73E-03
CCNB1	5.97E-05	EZRIN	3.35E-03	TAZ	5.10E-05
CDK1	1.05E-03	FN1	2.88E-03	TRAP1	4.09E-03
CDKN1B.pT198	1.16E-03	GAB2	1.54E-05	VTCN1	7.50E-03
CHEK1	4.03E-03	H2AX.p140	8.21E-04		
DNM1L	1.61E-06	HNRNPK	5.70E-03		
DNMT1	3.57E-03	ITGAL	1.14E-03		
DUSP4	8.41E-03	KAT2A	2.86E-03		
EIF2S1.pS51	1.92E-03	LGALS3	2.97E-03		
EIF4E	6.95E-04	MAPK14	4.08E-04		
EIF4G	1.59E-06	MAPK14.pT180.Y182	4.88E-04		
ELAVL1	6.52E-03	MSI2	1.57E-04		
ELK1.pS383	1.32E-04	NLN	5.38E-04		
ERCC1	5.89E-04	PAK1	9.04E-04		
EZH2	9.74E-06	PARP_cleaved	2.43E-06		
FASN	9.67E-03	PCNA	5.15E-03		
FLI1	3.44E-03	PECAM1	6.73E-03		
FN1	1.06E-04	PIM2	9.23E-04		
GLS	5.43E-03	PTEN	1.49E-03		
GYS1	4.35E-03	RAD50	6.41E-03		
H2AX.p140	6.93E-04	RPS6KB1.pT389	9.38E-03		
H3K27Me3	3.50E-05	SDHA	7.40E-04		
H3K36Me3	6.41E-04	SGK3	1.35E-05		
H3K4Me1	7.94E-04	SMAD5	2.37E-03		
HDAC2	4.43E-03	SOD1	1.92E-03		
HSPD1	2.36E-03	SPI1	2.78E-03		
JAK2	4.29E-03	SRC.1	1.47E-05		
KMT2A	9.72E-03	SRC.pY527	8.59E-03		
LDHA	3.15E-03	TFRC	1.16E-08		
MAP2K1.2.pS217.221	6.79E-03	TSC2	6.50E-03		
MAP2K2	1.62E-03	ZAP70	8.80E-08		
MAPK1.3.pT202.Y204	1.31E-04				
MCL1	3.03E-05				
MDM2.pS166	7.42E-03				
MEF2C	4.35E-04				
MEN1	4.38E-03				
MNK1	4.00E-13				
MUC1	1.22E-03				
NCSTN	8.07E-04				
NLN	9.59E-03				
NOTCH1_cleaved	1.53E-03				
NOTCH3	1.53E-03				
NUMB	1.23E-05				
PAK1	1.14E-04				
PARK7	5.23E-03				
PARP	5.81E-04				
PCNA	7.73E-03				
PDGFRB	7.08E-06				
PIM2	6.07E-04				
PKM	6.19E-03				
RPS6KB1.pT389	1.19E-03				
SF3B1	1.17E-04				
SGK3	2.88E-03				
SMAD2	6.36E-03				
SMAD2.p245.250.255	1.87E-03				
SOD1	1.08E-03				
SPI1	3.98E-05				
SPP1	2.83E-03				
SRSF1	1.80E-03				
SSBP2	1.74E-03				
STK11	3.30E-03				
TAZ	3.12E-03				
TRAP1	1.61E-03				
TUBA1A_Detyro	4.99E-03				
ZAP70	3.88E-05				

Supplementary Fig S4. Outcome based on SOD1 expression level.

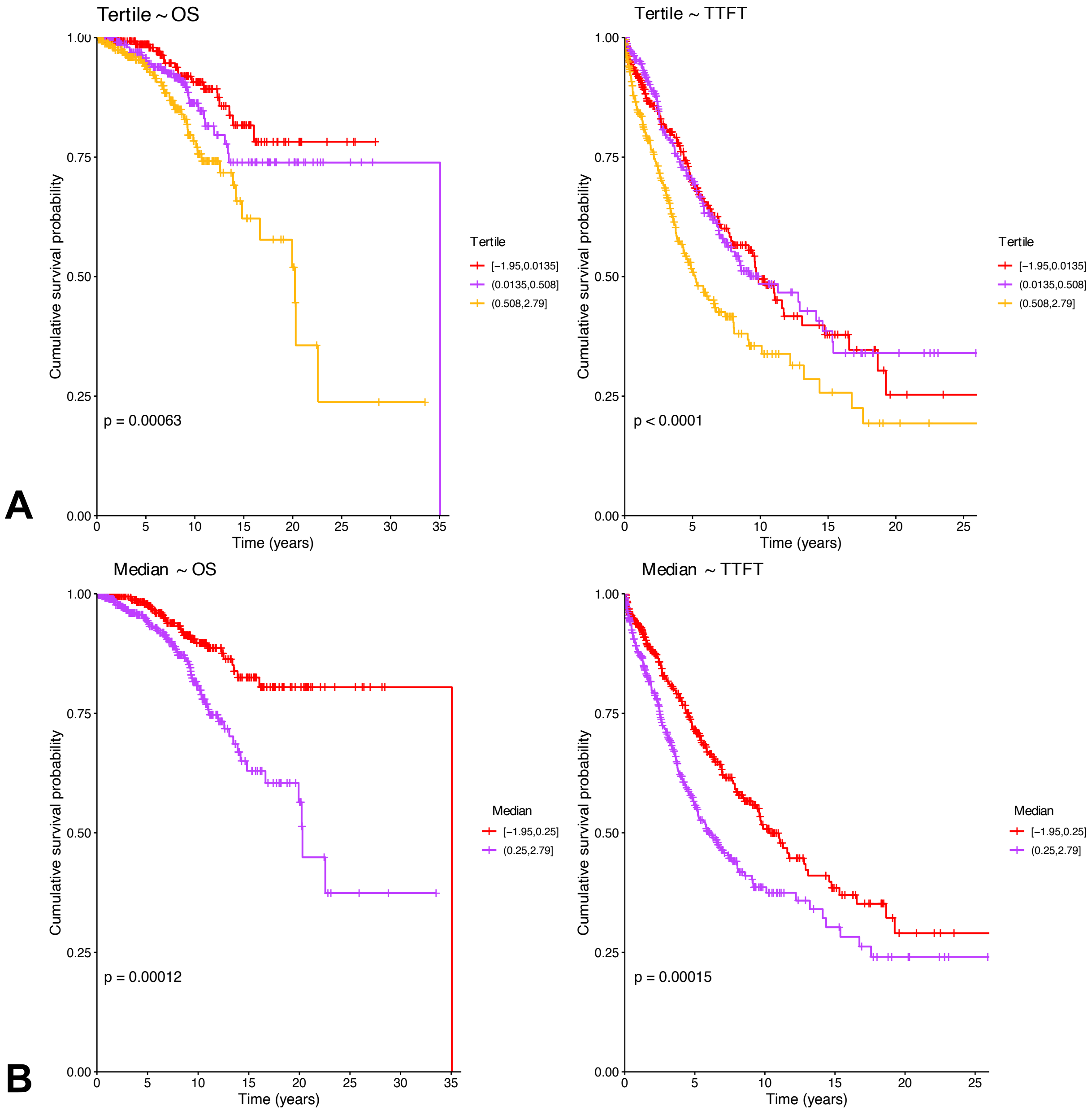

Supplementary Fig S5. Proteins Associated with BTKi Overall Survival.

Median (N=57)	Tertile (N=78)	Sextile (N=78)
ACACA.pS79	ACACA	ACTB
AKT1.2.3.pS473	ACTB	AKT1.2.3.pT308
AKT1.AKT2.AKT3	AKT1.2.3.pS473	AKT1S1.pT246
AKT3	AKT1.2.3.pT308	ASNS
ARID1A	AKT1.AKT2.AKT3	ATG3
ASNS	AKT3	ATG4B
ATM.pS1981	ASNS	ATM.pS1981
BMI1	ATG7	BAX
CD44	ATM.pS1981	BIRC5
CDH1	BRAF.pS445	BRAF.pS445
CDKN1B.pT157	BRD4	BRD4
CDX2	CASP7_cleaved	CASP7_cleaved
CHEK1	CBL	CCND3
CHEK1.pS296	CCND3	CD44
CHEK2.pT68	CD44	CD86
E2F1	CDH1	CDH1
EEF2	CDKN1B.pT198	CDKN1B.pT198
EIF4E	CDX2	CDX2
EIF4EBP1.pS65	CHEK1	CHEK1
FLI1	CHEK2.pT68	CREB
GSK3A.B	CREB.1	CTSG
H2AX.p140	DDX17	DDX17
HIF1A	DUSP4	DLX1
IGF1R.pT1135.T1136	E2F1	DUSP4
JAK2	EEF2	EEF2K
KDR	EIF4E	EIF4E
KMD1A	EIF4EBP1.pS65	EIF4G2
KMT2D	EIF4G2	ELK1.pS383
LEF1	ELK1.pS383	EP300
LYN	EP300	FN1
MAPK8.pT183.p185	FLI1	FOXO3.pS318.S321
MAPK9	FN1	GAPDH
MDM2.pS166	GAPDH	GLS
MDM4	GLUD	GLUD
MEF2C	GSK3A.B	GSK3A.B
NCSTN	H2AX.p140	H2AX.p140
NUMB	HDAC2	HSF1
PAK1	HIF1A	HSPA9
PDGFRB	HSPA9	KDR
PIM2	IGFR1	KMT2D
PRKAA1.2.pT172	KDR	LEF1
PRKCA	KMD1A	LYN
RELA.pS536	KMT2D	MAP2K1.2.pS217.221
RPA2	LEF1	MAPK1.3.pT202.Y204
SF3B1	LYN	MAPK9
SGK3	MAP2K1.2.pS217.221	MDM4
SMAD1	MAPK1.3.pT202.Y204	MEF2C
SRSF1	MAPK9	MEN1
SSBP2	MDM2.pS166	MNK1
STAT5A	MDM4	MSH6
TP53BP1	MEF2C	MTOR
TSC2	MEN1	MYH11
VASP	MTOR	NCL
VCP	MUC1	NCSTN
WEE1	MYH11	NUMB
YAP	NCSTN	PARP
ZAP70	NOTCH2	PDGFRB
	NUMB	PRKCA
	PARP	RPA2
	PARP_cleaved	RPS6.p240.244
	PDGFRB	SDHA
	PIM1	SETD1A
	PLK1	SF3B1
	PRKAA1.2.pT172	SMAD1
	RELA.pS536	SOD2
	RPA2	SPARC
	RPS6.p240.244	SPI1
	S100A4	STK11
	SDHA	SUZ12
	SF3B1	TFRC
	SMAD1	TP53BP1
	SMAD2	VASP
	SPI1	VIM
	TFRC	VTCN1
	TP53BP1	WDR5
	VASP	WTAP
	WTAP	YAP
	YAP	YAP1.pS127

Supplementary Fig S6. Protein Functional Group Expression Patterns present in CLL and CD19+ controls.

		CD19				
		Protein Cluster				
PFG	N	1	2	3	4	5
Adhesion	4	Checkered	Solid	Solid	Checkered	
Apoptosis_BH3	4	Solid	Solid	Checkered	Solid	
Apoptosis_Occuring	4	Solid	Solid	Checkered	Solid	
Apoptosis_Regulating	3	Checkered	Solid	Checkered		
Autophagy	4	Checkered	Solid	Solid	Checkered	
BCR	3	Solid	Solid	Checkered		
Cell_Cycle	5	Solid	Solid	Checkered	Solid	Checkered
CREB	3	Solid	Checkered	Solid		
Cytoskeletal	5	Checkered	Solid	Solid	Solid	Solid
Differentiation	4	Solid	Checkered	Solid	Solid	
DNA_damage	3	Checkered	Solid	Solid		
GPCR	3	Checkered	Solid	Solid		
Heatshock	4	Solid	Checkered	Checkered	Solid	
Hippo	4	Checkered	Checkered	Checkered	Solid	
Histone1	4	Solid	Checkered	Checkered	Solid	
Histone2	3	Solid	Checkered	Checkered		
Hypoxia	3	Solid	Solid	Checkered		
MAPK	4	Checkered	Checkered	Solid	Solid	
MEK_RAS	4	Solid	Checkered	Solid	Solid	
Metabolic_FAS	4	Checkered	Checkered	Solid	Solid	
Metabolic_Glucose	4	Solid	Checkered	Checkered	Solid	
Metabolic_Lipid	3	Checkered	Checkered	Solid		
Metabolic_OR	4	Checkered	Solid	Solid	Solid	
Metabolic_Protein	4	Checkered	Solid	Checkered	Solid	
MTOR	3	Checkered	Solid	Solid		
Phosphatase	4	Checkered	Solid	Solid	Solid	
PI3KAKT	4	Solid	Checkered	Checkered	Checkered	
PKC	5	Solid	Solid	Solid	Solid	Solid
Ribosomal_Activity	4	Solid	Solid	Checkered	Solid	
RNA_splicing	4	Solid	Checkered	Solid	Checkered	
SMAD	3	Solid	Solid	Checkered		
SRC	4	Solid	Checkered	Solid	Solid	
STAT	4	Checkered	Checkered	Solid	Solid	
STP_Regulation	3	Checkered	Checkered	Solid		
T_Cell	3	Solid	Checkered	Solid		
TP53	3	Checkered	Solid	Solid		
Transcription	4	Solid	Solid	Checkered	Solid	
Ubiquitin	4	Solid	Solid	Solid	Solid	
UPR	3	Solid	Solid	Checkered		
Wnt_signaling	5	Checkered	Solid	Solid	Solid	Checkered

Solid: Leukemia Specific	Solid
Checkered: Control-like	Checkered

Supplementary Fig S7: Protein Function Groups with a Single Prognostic Cluster.

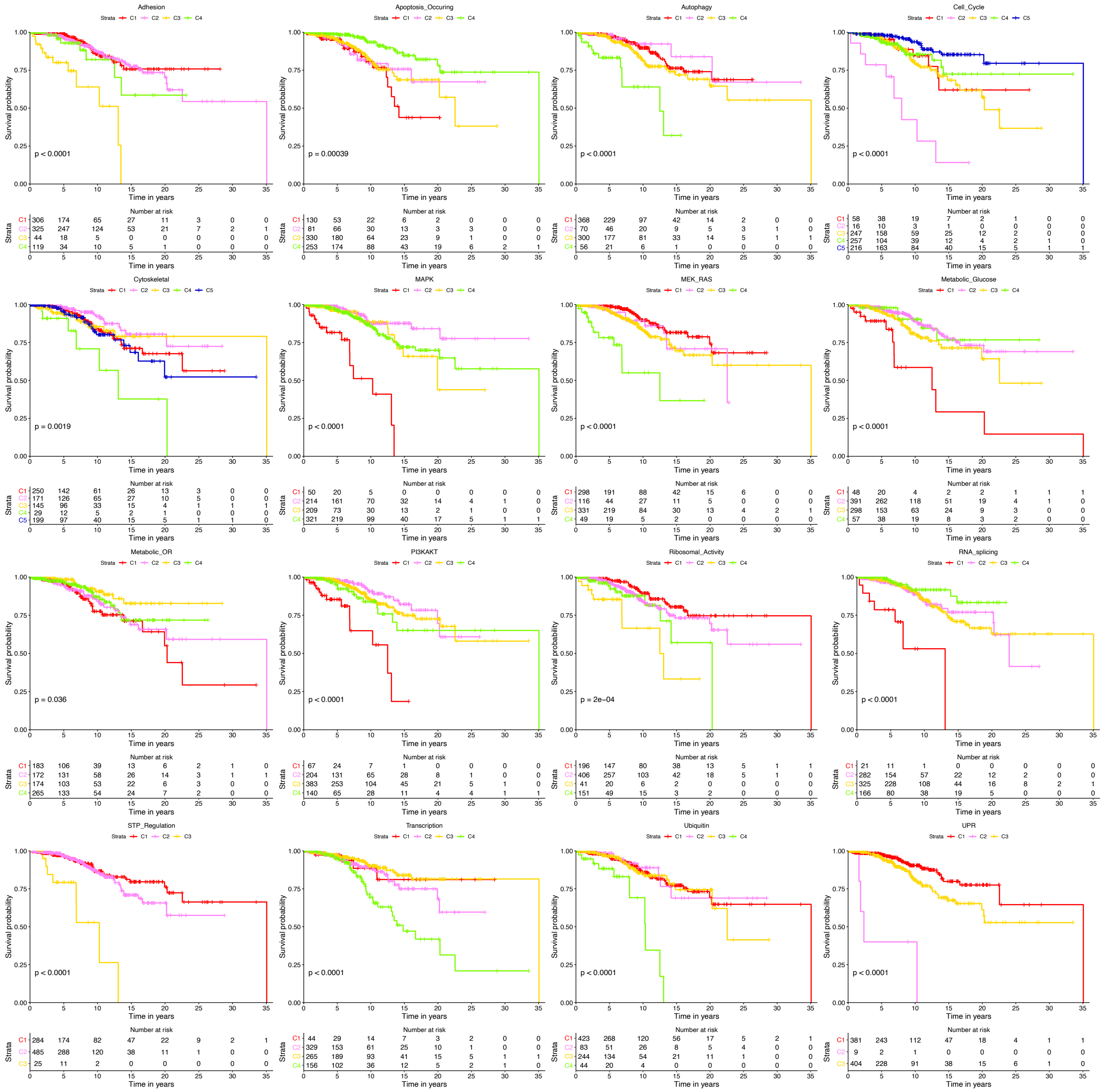

Supplementary Fig S8: Protein Function Groups with Heterogenous Outcomes.

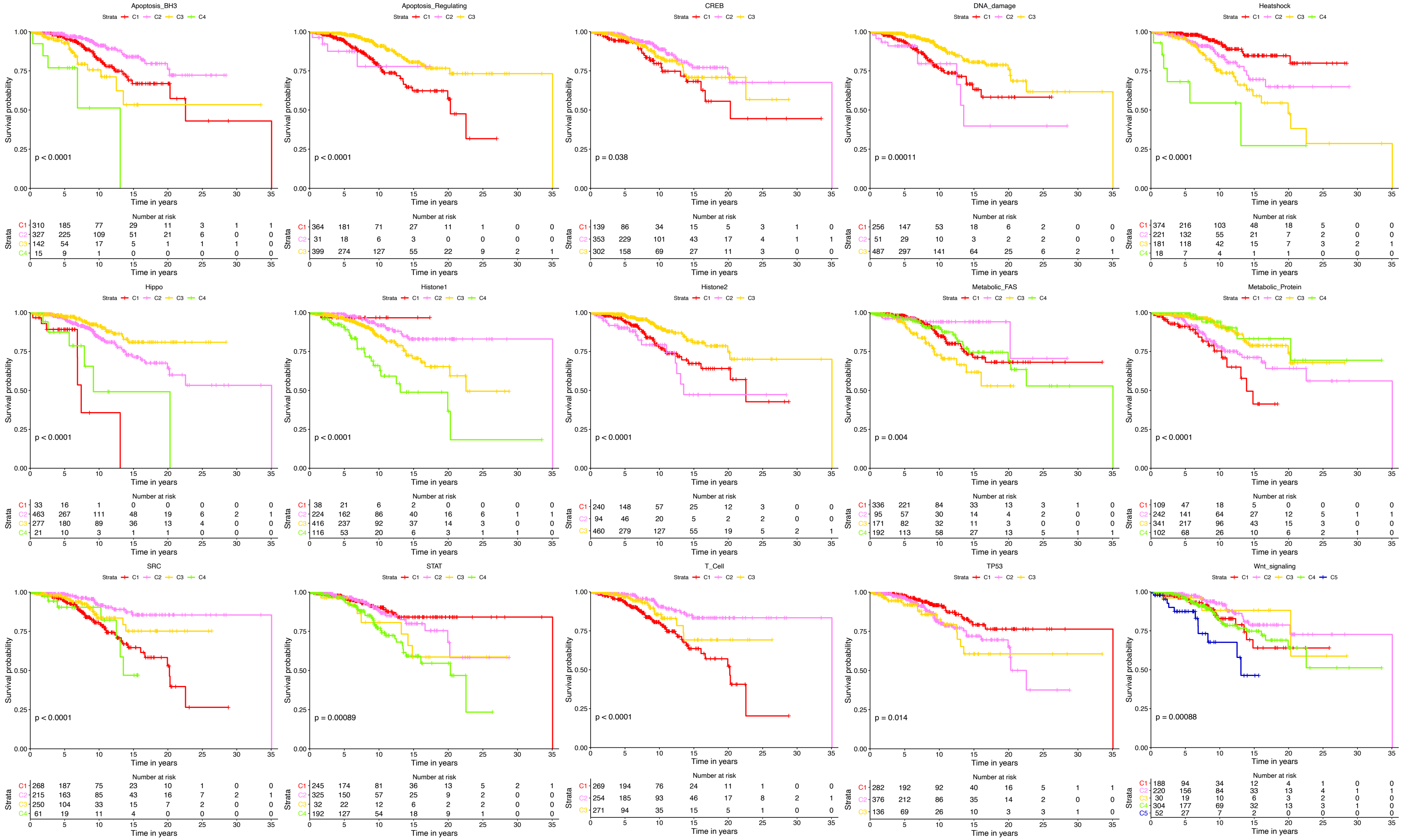

Supplementary Fig S9: Protein Function Groups that were not Prognostic.

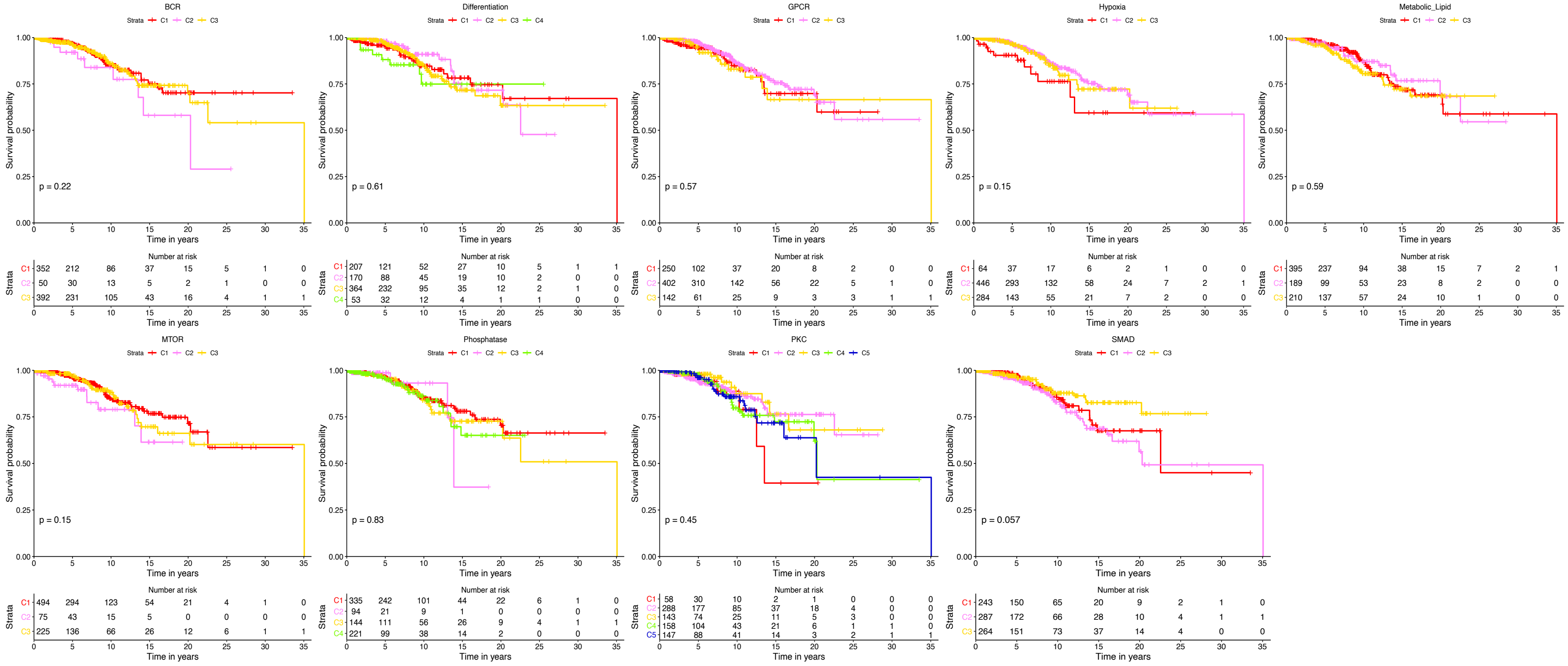

Supplemental Figure S10: Overall Survival of CLL Numeric Signatures

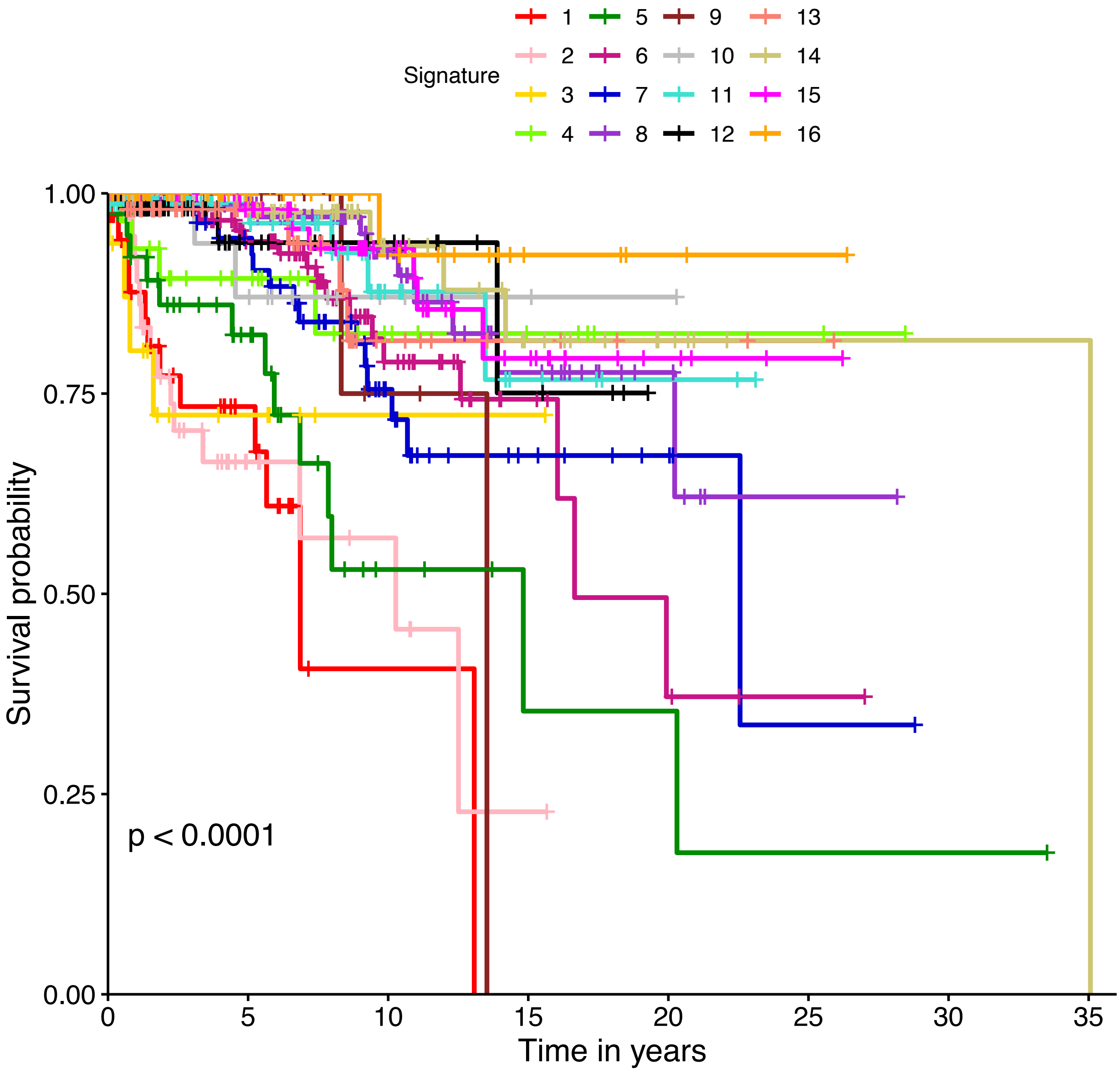

Supplemental Fig S11: CLL Signature Group Lab Test Boxplots

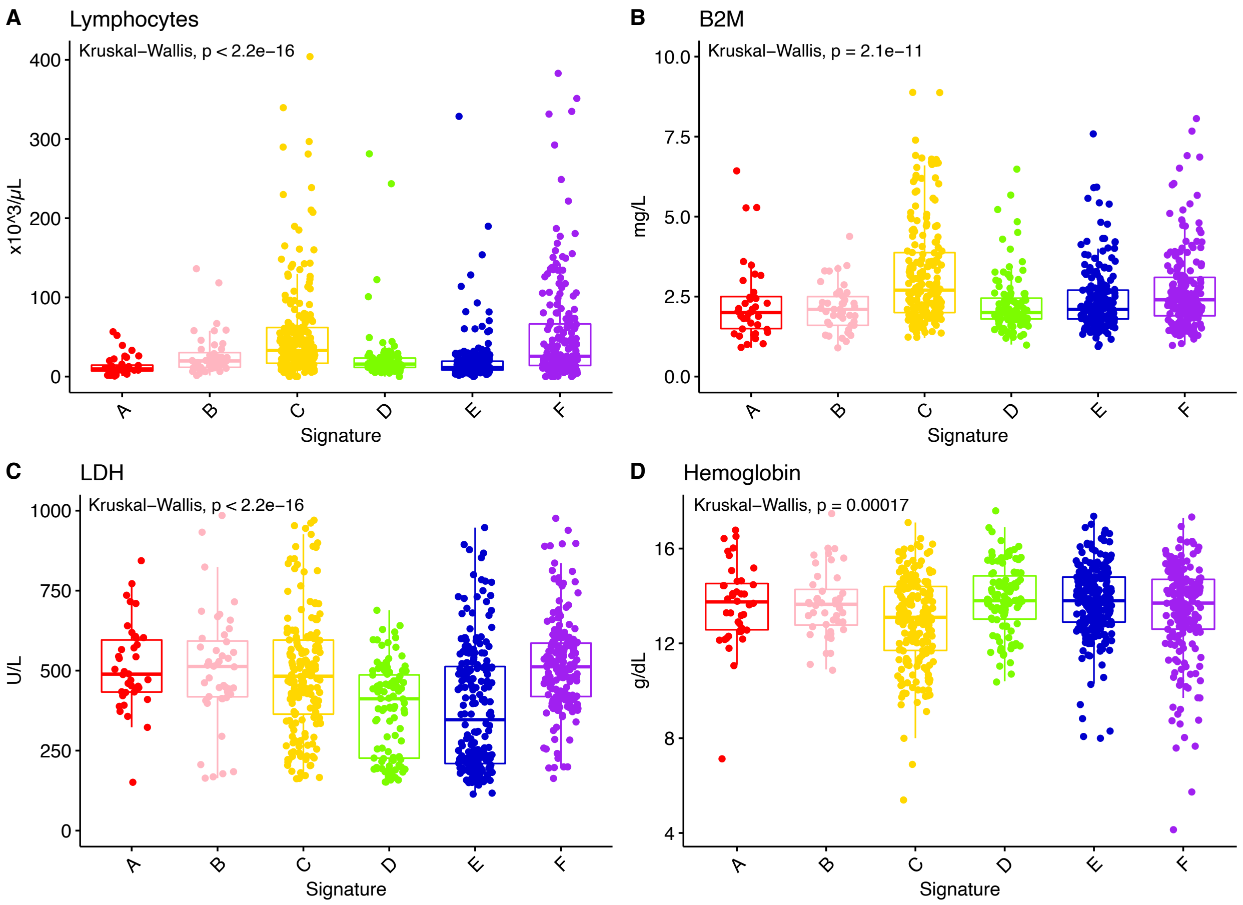

Supplementary Fig S12: Signature Group outcomes stratified by Rai stage.

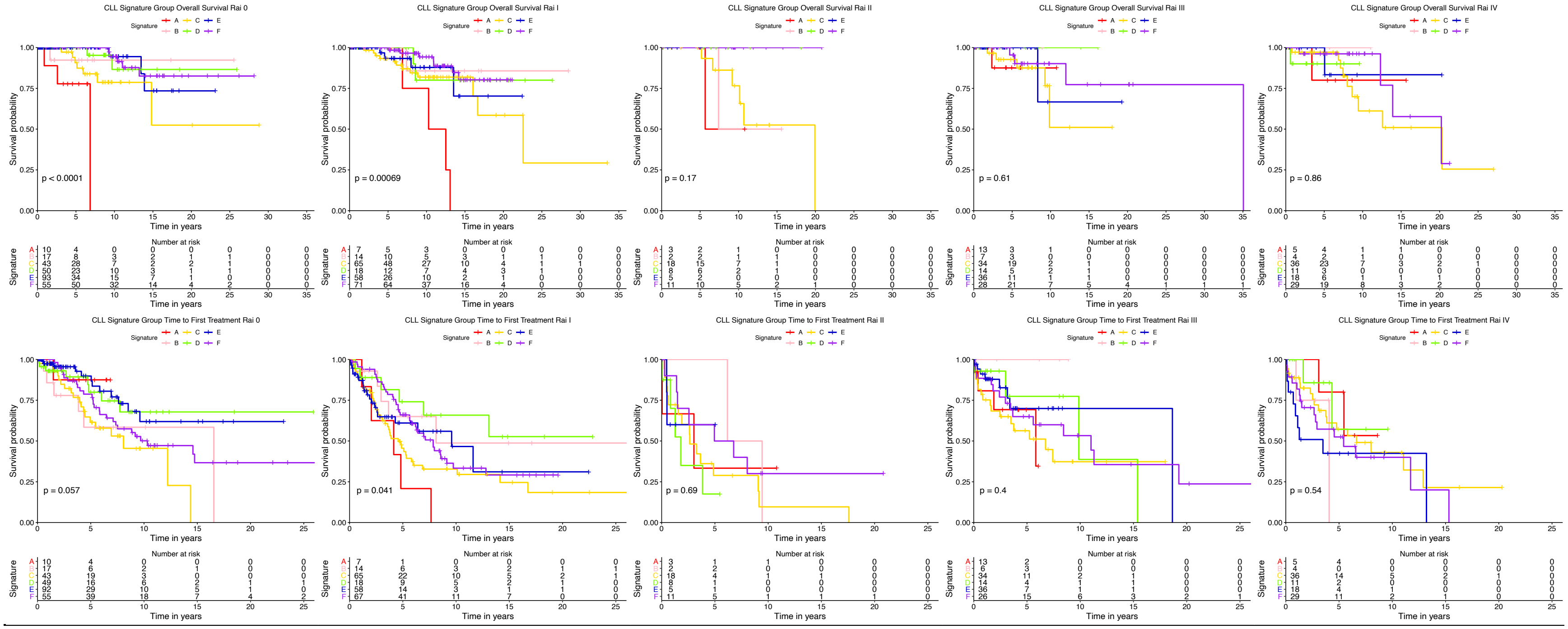

Supplementary Fig S13: Overall survival of Rai stages stratified by signature group.

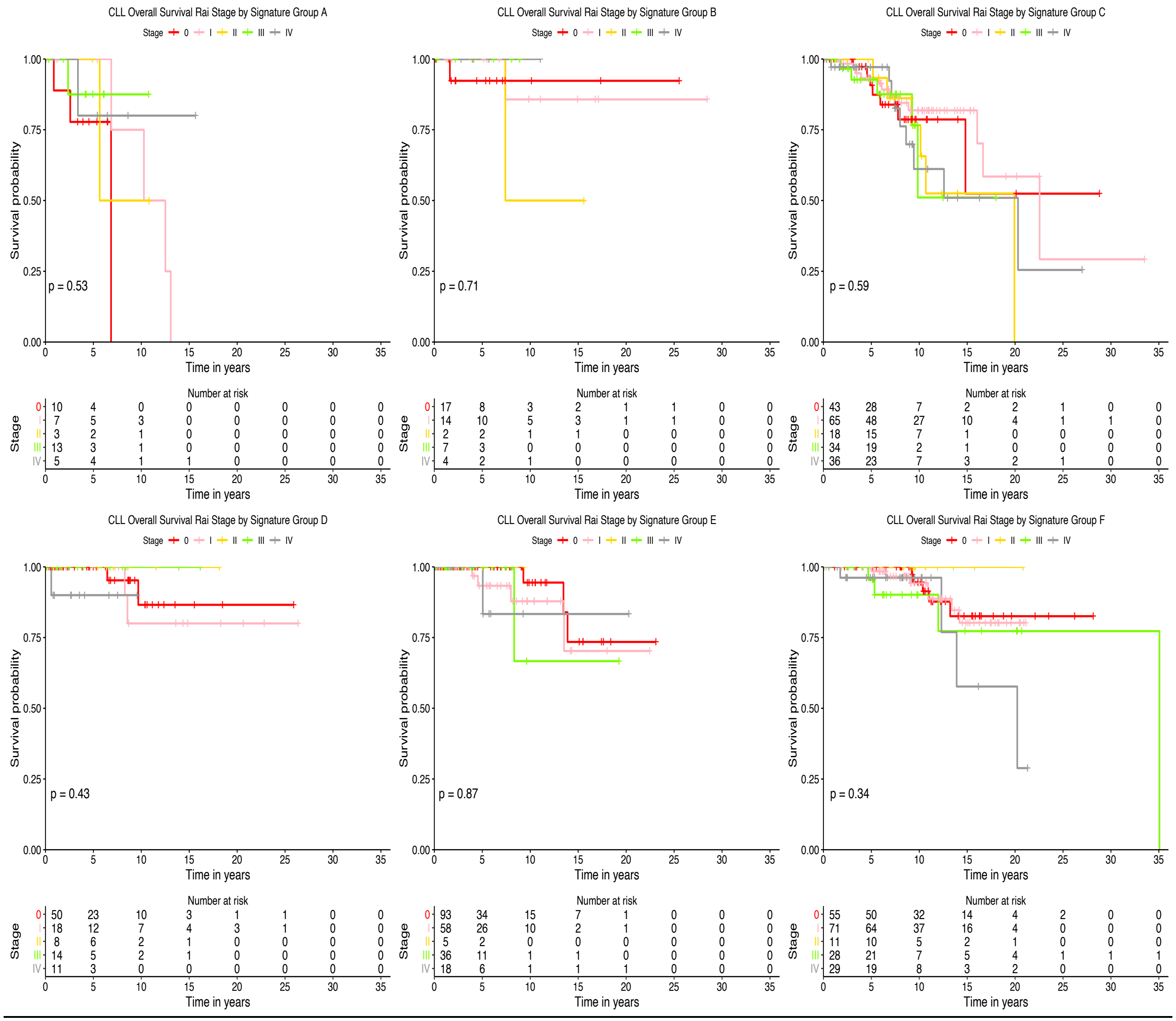

Supplementary Fig S14: OS, TTFT, and TTST of IGHV Status Within Signature Groups.

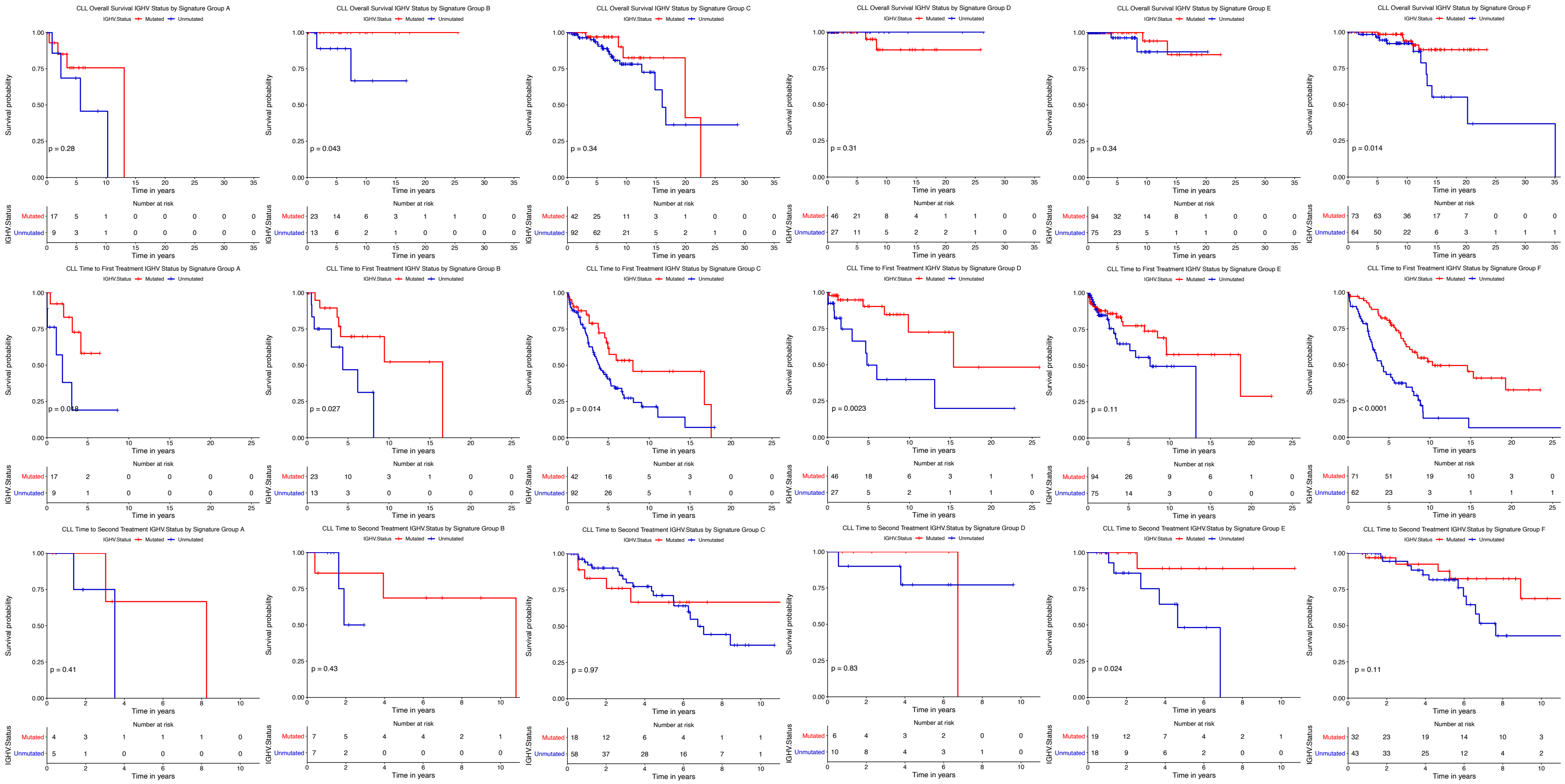

Supplementary Fig S15: CLL-IPI Classification Group outcomes stratified by Signature Group.

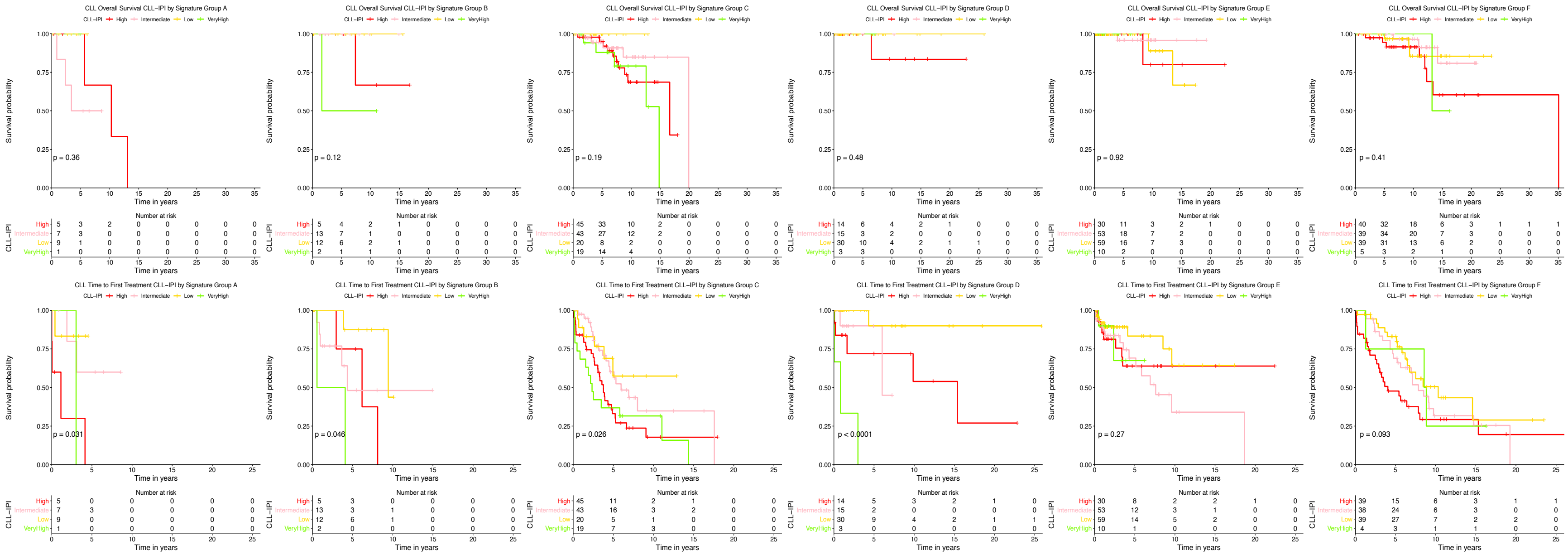

Supplementary Fig S16: Signature Group outcomes stratified by CLL-IPI Classification Group.

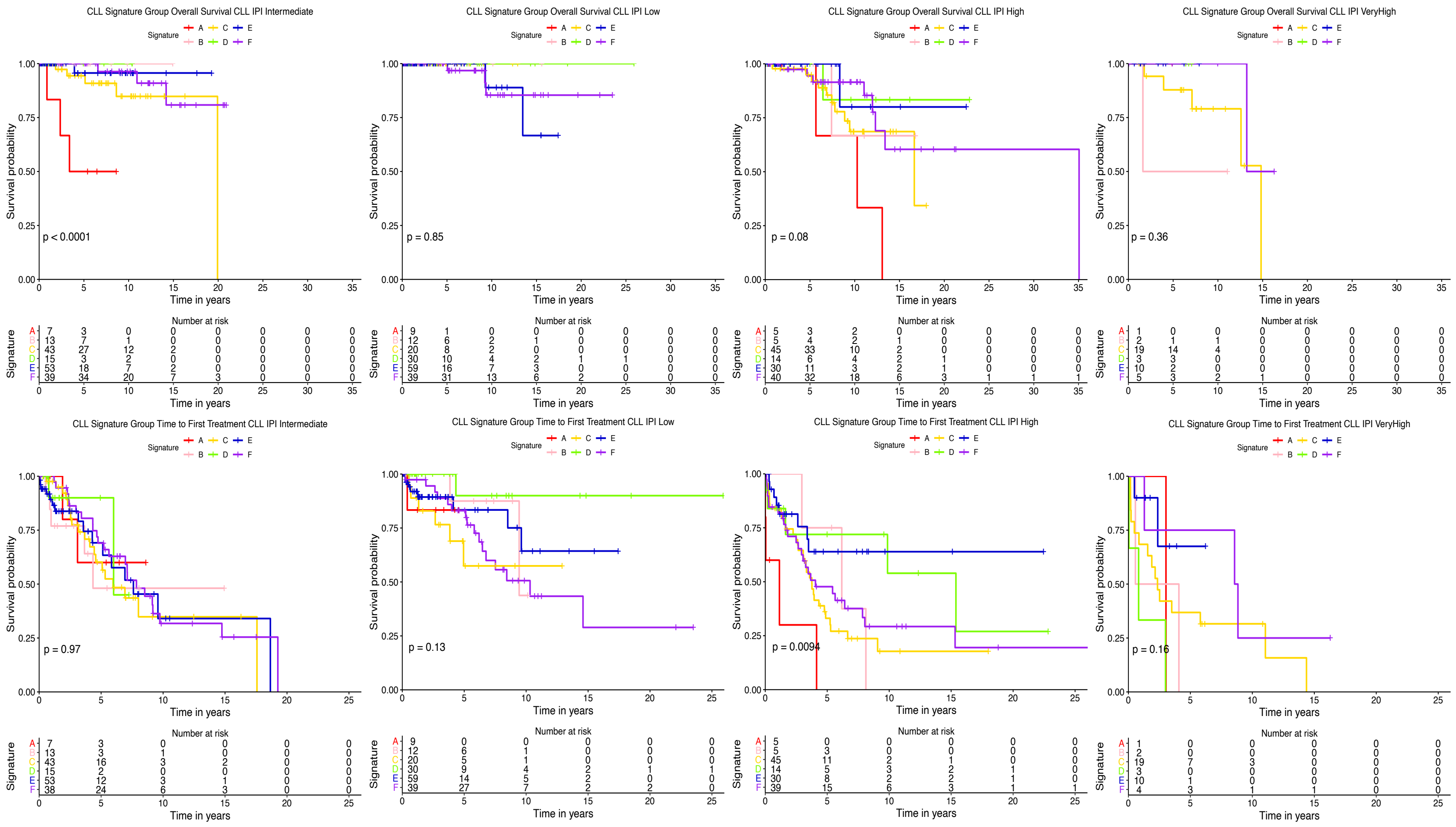

Supplementary Fig S17: Signature Group outcomes stratified by 17p and 11q deletion grouping.

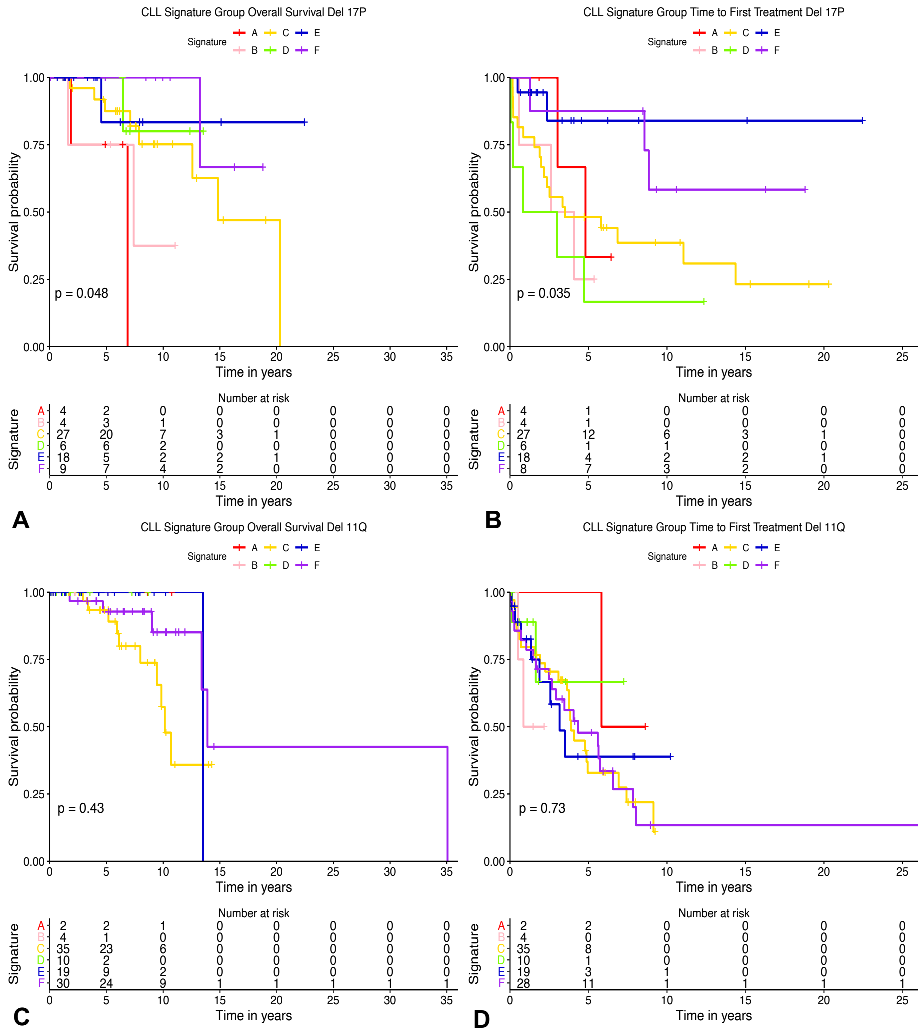

Supplementary Fig S18. Heatmap of Metabolic Glucose Protein Functional Group Members.

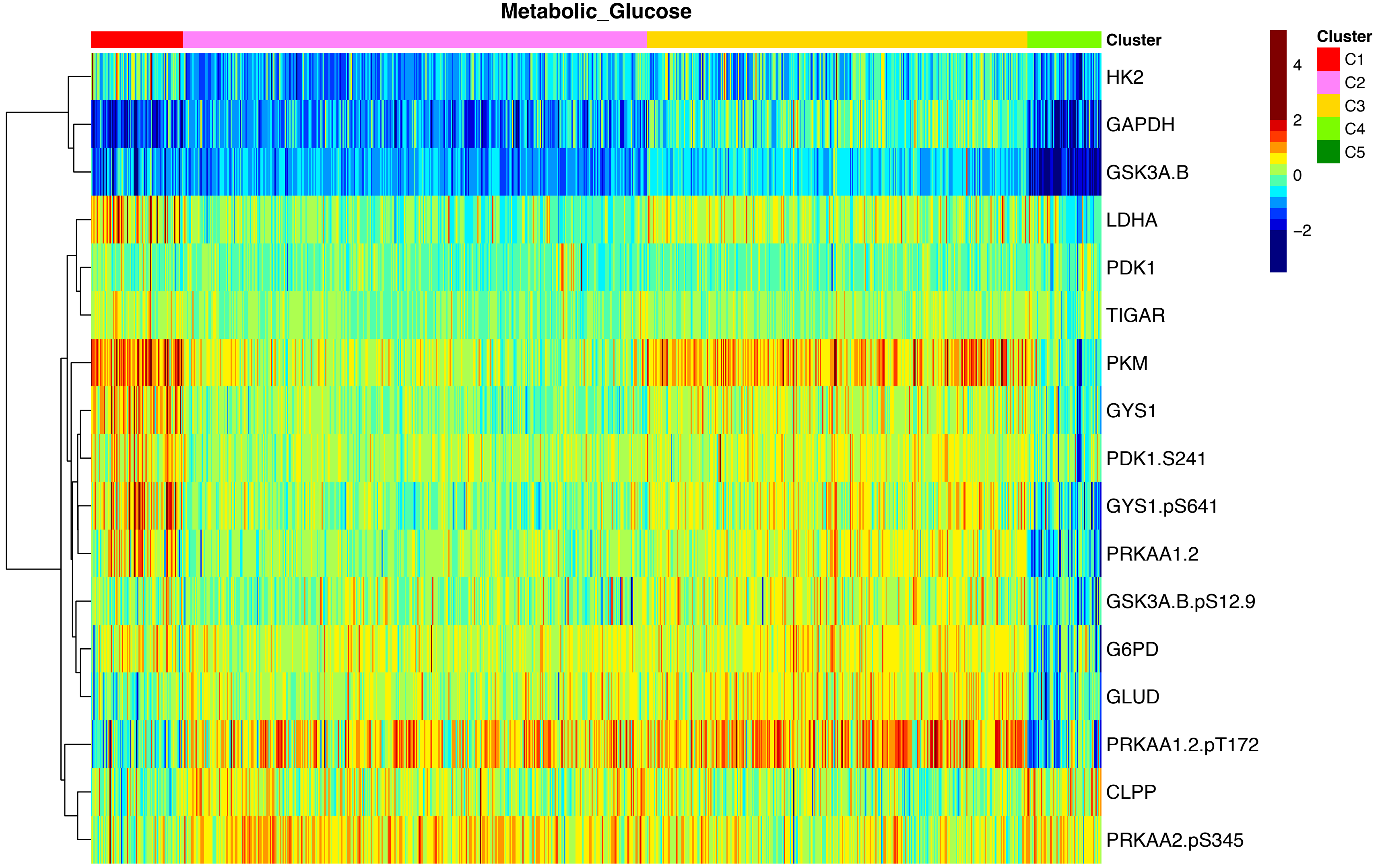

Supplementary Fig S19: Heatmap and Dot plots of Discriminative Proteins.

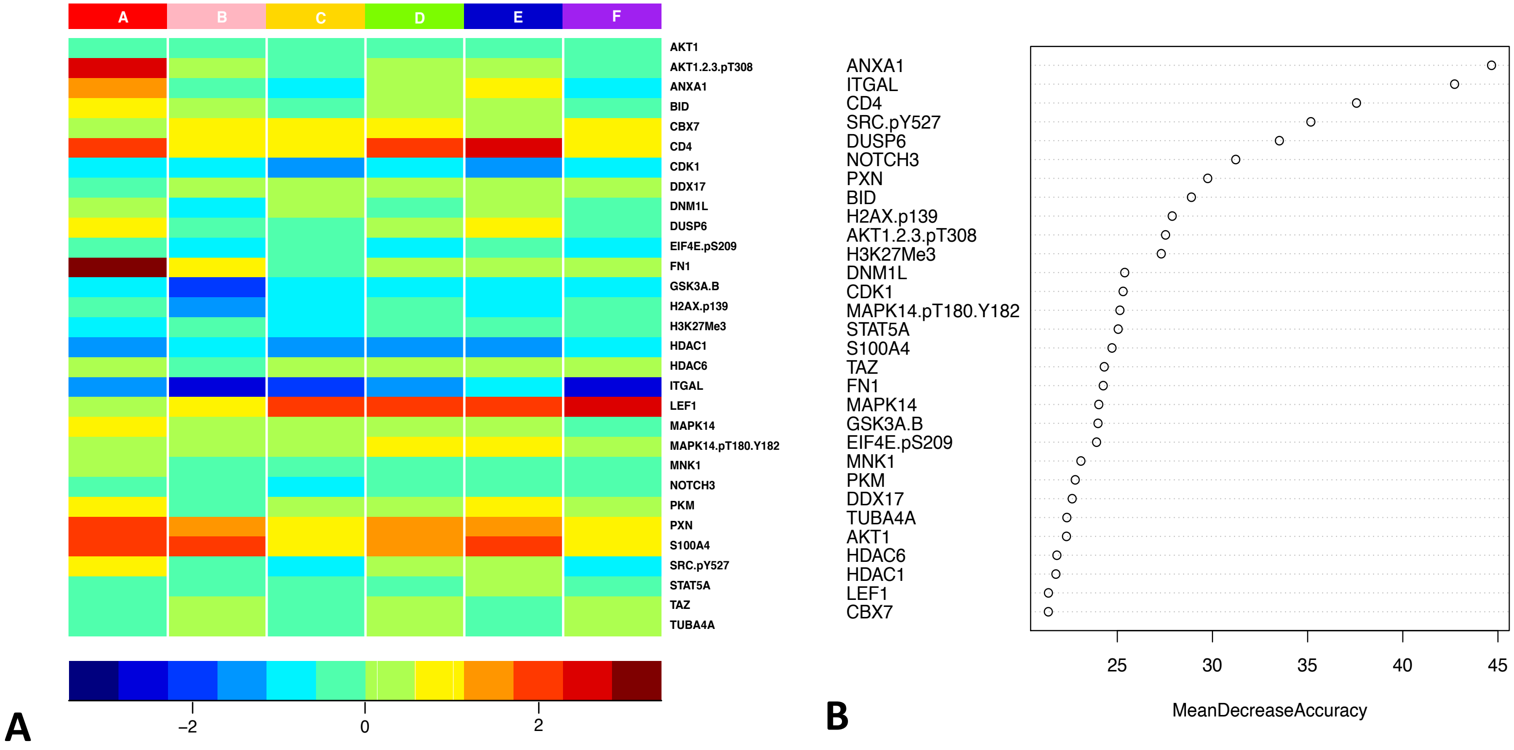

Supplementary Fig S20: Overall Survival of Classified and Misclassified SG-A

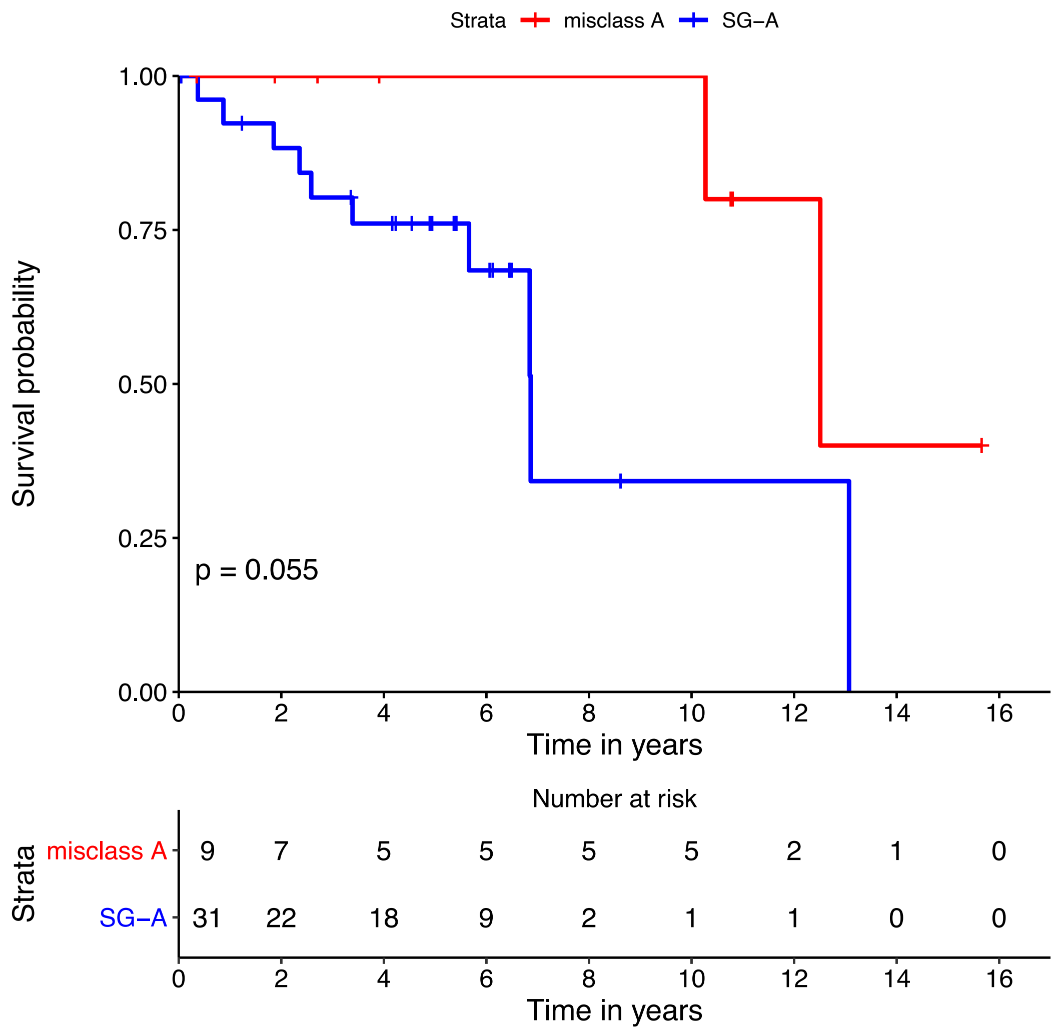

Supplemental Fig S21: Overall Survival of Misclassified Signature Group Members

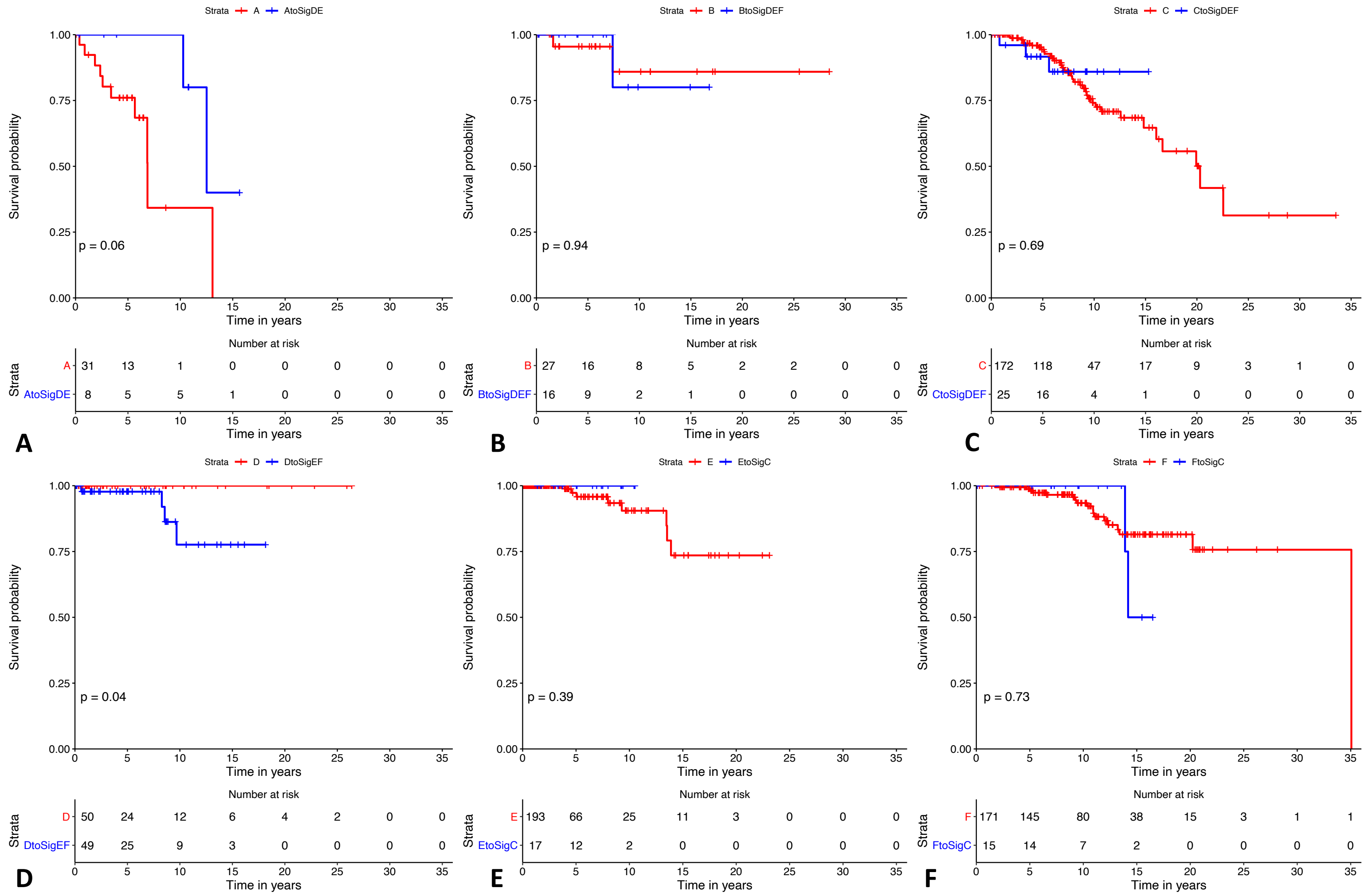

Supplementary Fig S22: RPPA Sample Purity Boxplot

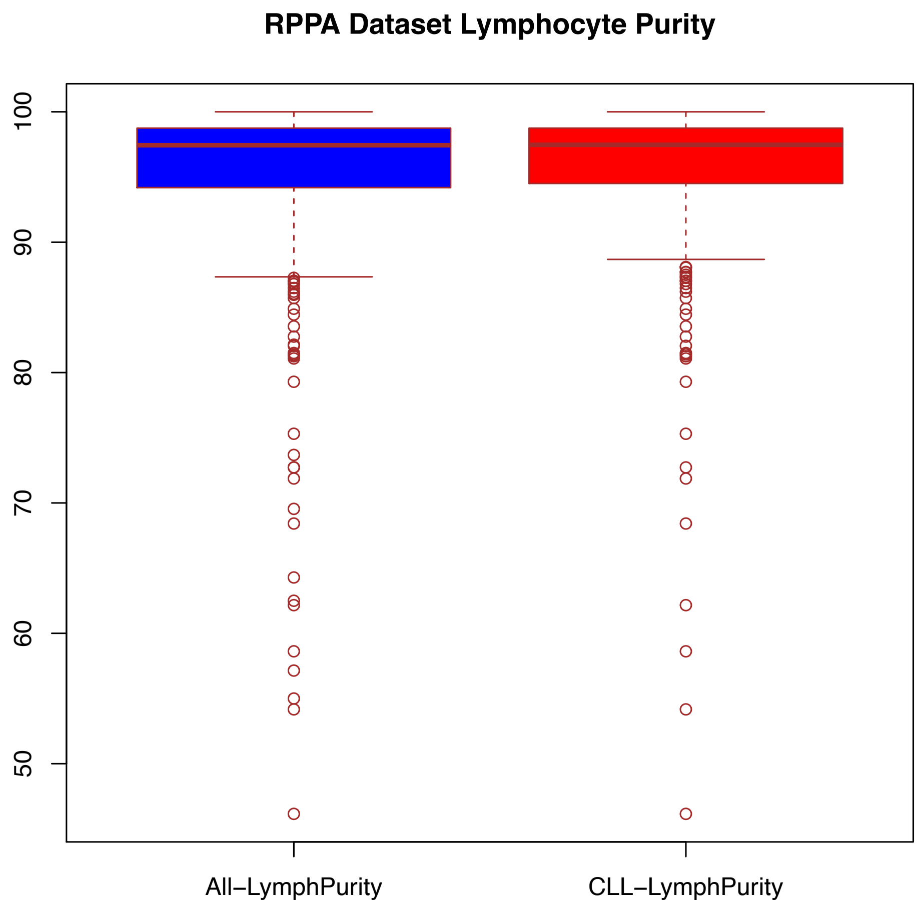

Supplement: Supplementary file 1 — Supplemental Figures and Legends [file 41408_2022_623_MOESM1_ESM.pdf]
