# Supplementary material for: Proteomic profiling based classification of CLL provides prognostication for modern therapy and identifies novel therapeutic targets
Source: Blood Cancer J. 2022 Mar 17;12(3):43. doi: 10.1038/s41408-022-00623-7 (PMC8931092; doi:10.1038/s41408-022-00623-7)
Supplement: Supplementary file 3 — Supplementary Table 2 - Table of Protein Functional Groups and Protein Members [file 41408_2022_623_MOESM3_ESM.docx]

| **PFG** | **N** | **Proteins** |
| --- | --- | --- |
| Adhesion | 15 | CD44,CDH1,CDH2,EZRIN,FN1,ITGA2,ITGAL,ITGB1,  LMNB1,MMP2,MUC1,PECAM1,PXN,SPP1,VASP |
| Apoptosis  BH3 | 11 | BAK,BAX,BBC3,BCL2L1 ,BCL2,BCL2.pS70,BCL2A1,  BCL2L11,BID,MCL1,TGM2 |
| Apoptosis  Occurring | 7 | CASP3,CASP3_cleaved,CASP7_cleaved,CASP9,  PARP,PARP_cleaved,RIPK1 |
| Apoptosis  Regulating | 9 | AIFM1,BIRC2,BIRC3,BIRC5,DNM1L,NOL3,TNFRSF4,  XIAP,LMNB1 |
| Autophagy | 12 | ATG3,ATG4B,ATG7,BECN1,PARK7,SQSTM1,STK11,  ULK1.pS757,KEAP1,PRKAA1.2,PRKAA1.2.pT172,  PRKAA2.pS345 |
| BCR | 4 | BTK,SYK,CBL,LYN |
| Cell Cycle | 30 | AURKB,CCND1,CCND3,CCNE1,CDC25C,CDK1,  CDK1.pY15,CDK2,CDKN1A,CDKN1B,CDKN1B.pS10,  CDKN1B.pT157,CDKN1B.pT198,CDKN2A,CSNK2A1,  E2F1,FOXM1,FOXO3,FOXO3.pS318.S321,FZR1,  MYC,PCNA,PLK1,RB1,S100A4,SIRT1,SPARC,UGT1A,  WEE1,WEE1.pS642 |
| CREB | 3 | ATF3,CREB,CREB1.pS133 |
| Cytoskeletal | 11 | ACTB,ANXA7,MYH11,MYH9.pS1943,PAK4,  RAB11A.11B,STMN1,  TUBA1A_Detyro,TUBA4A,VIM,AURKB |
| Differentiation | 5 | GATA1,GATA3,PIM1,PIM2,SOX2 |
| DNA damage | 24 | ATM,ATM.pS1981,BRCA2,CHEK1,CHEK1.pS296,  CHEK1.pS345,CHEK2,CHEK2.pT68,DDB1,ERCC1,  ERCC5,MSH2,MSH6,PDCD1,RAD50,  RAD51,RPA2,RPA2.pS4.8,SSBP2,TP53BP1,  VCP,XPA,XPF,XRCC1 |
| GPCR | 9 | ANXA1,STK4,NLN,PREX1,PTK2,PTK2.pY397,  CXCR5,DNM1L,EZRIN |
| Heatshock | 9 | HSF1,HSF1.pS326,HSP90AA1.HSP90AB1,HSPA1A,  HSPA5,HSPA9,HSPB1.pS82,HSPD1,TRAP1 |
| Hippo | 8 | LATS1,NF2,TAZ,YAP,YAP1.pS127,BIRC5,STK4,STK11 |
| Histone1 | 21 | BRD4,CBX7,DNMT1,EZH2,H2AX.p139,H2AX.p140,  H3K27Me3,H3K36Me3,H3K4Me1,H3K4Me2,H3K4Me3,  H3K9Me2,HDAC1,HDAC2,HDAC3,HDAC6,HIST3H3,  KAT2A,MEN1,SETD1B,XPO1 |
| Histone2 | 15 | ASH2L,BMI1,HIST1H2B.Ub,HNRNPK,JMJD6,KMD1A,  KMT2A,KMT2D,NCL,NPM1_C,PAK1,SETD1A,  SUZ12,WDR5,WTAP |
| Hypoxia | 6 | EGLN1,EP300,HIF1A,KDR,KDR.pY117.5,VHL |
| MAPK | 20 | COG3,CTSG,MAP2K1,MAP2K1.2.pS217.221,  MAP2K1.MAP2K2,MAP2K2,MAPK1,MAPK1.3.pT202.Y204,  MAPK14,MAPK14.pT180.Y182,MAPK8.MAPK10,  MAPK8.pT183.p185,MAPK9,MNK1,NUMB,PTPN11  ,PTPN11.pY542,DUSP4,DUSP6,FN1 |
| MEK_RAS | 11 | ARAF,BRAF,BRAF.pS445,ELK1.pS383,MET.pY1234.Y1235,  NRAS,RAB25,RAF1,RHEB,TNK1,YWHAE |
| MetabolicFAS | 4 | ACACA,ACACA.pS79,FASN,SCD |
| Metabolic Glucose | 17 | CLPP,G6PD,GAPDH,GLUD,GSK3A.B,GSK3A.B.pS12.9,GYS1  ,GYS1.pS641,HK2,LDHA,PDK1,PDK1.S241,PKM,PRKAA1.2,  PRKAA1.2.pT172,PRKAA2.pS345,TIGAR |
| Metabolic  Lipid | 2 | PPARA,PPARG |
| Metabolic  OR | 5 | AKR1C3,NDUFB4,SDHA,SOD1,SOD2 |
| Metabolic  Protein | 3 | ASNS,ASS1,GLS |
| MTOR | 9 | MTOR,MTOR.pS2448,RICTOR,RICTOR.pT1135,  RPTOR,TSC1,TSC2,TSC2.pT1462,AKT1S1.pT246 |
| Phosphatase | 2 | DUSP4,DUSP6 |
| PI3KAKT | 14 | AKT1,AKT1.2.3.pS473,AKT1.2.3.pT308,AKT1.AKT2.AKT3,  AKT1S1.pT246,AKT2,AKT3,CXCR5,IGFR1,INPPL1,PIK3CA,P  IK3CB,PTEN,TYRO3 |
| PKC | 5 | PRKAR1A,PRKCA,PRKCA.pS657,PRKCB.pS660,PRKCD.pS664 |
| Ribosomal  Activity | 22 | DDX17,EEF2,EEF2K,EIF2AK2,EIF2S1,EIF2S1.pS51,EIF4E,  EIF4E.pS209,EIF4EBP1,EIF4EBP1.pS65,EIF4G,EIF4G2,  ELAVL1,MSI2,NDRG1.pT346,PDCD4,RPS6,  RPS6.p235.236,RPS6.p240.244,  RPS6KA1.pT573,RPS6KB1,RPS6KB1.pT389 |
| RNA  splicing | 5 | RPS6KA1.2.3,SF3B1,SF3B1.pT313,SRSF1,YBX1.pS102 |
| SMAD | 7 | SMAD1,SMAD2,SMAD2.p245.250.255,SMAD2.p465.467,  SMAD3,SMAD4,SMAD5 |
| SRC | 4 | LCK,LYN,SRC,SRC.pY527 |
| STAT | 7 | JAK2,SOCS2,STAT1,STAT3,STAT3.pS727,STAT3.pY705,S  TAT5A |
| STP  Regulation | 23 | ABL1,AXL,EGFR,EGFR.pY1173,EPHA2,EPHA2.pS897,  EPHA2.pY588,ERBB2,ERBB2.pY1248,GAB2,GAB2.pY452,  IGF1R.pT1135.T1136,IGFBP2,KIT,PDGFRB,SGK1,SGK3,SHC1.pY317,TFRC,  PTK2,PTK2.pY397,PTPN11,PTPN11.pY542 |
| T_Cell | 17 | CD276,CD4,CD74,CD86,JAG1,LGALS3,NOTCH1,NOTCH1_cleaved,  NOTCH2,NOTCH3,TAPBP,VTCN1,ZAP70,GATA3,LCK,PDCD1,TNFRSF4 |
| TP53 | 7 | MDM2,MDM2.pS166,MDM4,TP53,TIGAR,TP53BP1,YWHAE |
| Transcription | 16 | ARID1A,CDX2,SPI1,DLX1,ERG,ETS1,FLI1,HES1,HEXIM1,JUN.pS73,  JUNB,MEF2C,NFE2L2,RELA,RELA.pS536,YBX1.pS102 |
| Ubiquitin | 4 | CBL,COPS5,KEAP1,PSMB9 |
| UPR | 4 | ERN1,EIF2S1,EIF2S1.pS51,HSPA5 |
| Wnt  signaling | 12 | CAV1,CTNNB1,CTNNB1.pS33.S37.T41,CTNNB1.pT41.S45,DVL3,  LEF1,LRP6.pS1490,NCSTN,PTGS2,GSK3A.B,GSK3A.B.pS12.9,XPO1 |
