# Supplementary material for: Proteomic profiling based classification of CLL provides prognostication for modern therapy and identifies novel therapeutic targets
Source: Blood Cancer J. 2022 Mar 17;12(3):43. doi: 10.1038/s41408-022-00623-7 (PMC8931092; doi:10.1038/s41408-022-00623-7)
Supplement: Supplementary file 4 — Supplementary Table 3 - Table of Constellations and their Protein Functional Group Expression pattern membership [file 41408_2022_623_MOESM4_ESM.docx]

| Constellation | N | PFG Expression Patterns |
| --- | --- | --- |
| 1 | 22 | Adhesion_3,Apoptosis_BH3_4,Apoptosis_Regulating_2,Autophagy_4,BCR_2,Cell_Cycle_2,Cytoskeletal_4,DNA_damage_2,Heatshock_4,Hippo_1,Histone2_2,Hypoxia_1,MAPK_1,MEK_RAS_4,Metabolic_Glucose_1,MTOR_2,PI3KAKT_1,Ribosomal_Activity_3,RNA_splicing_1,STP_Regulation_3,Ubiquitin_4,UPR_2 |
| 2 | 10 | Adhesion_4,Apoptosis_BH3_3,Apoptosis_Occuring_1,Histone1_4,Metabolic_Protein_1,Phosphatase_2,PKC_1,SRC_4,TP53_3,Wnt_signaling_5 |
| 3 | 11 | Autophagy_2,Cell_Cycle_1,Differentiation_4,Hippo_4,Histone1_1,Metabolic_FAS_2,Metabolic_Glucose_4,Metabolic_Protein_4,STAT_3,Transcription_1,Wnt_signaling_3 |
| 4 | 7 | Cell_Cycle_4,GPCR_1,Hypoxia_3,Phosphatase_4,RNA_splicing_2,SRC_3,T_Cell_3 |
| 5 | 16 | Apoptosis_BH3_1,Apoptosis_Occuring_3,Apoptosis_Regulating_1,Autophagy_3,CREB_3,DNA_damage_1,Hippo_2,Histone1_3,MEK_RAS_3,Metabolic_Glucose_3,MTOR_1,PI3KAKT_3,STAT_2,TP53_2,Transcription_2,Wnt_signaling_4 |
| 6 | 10 | Differentiation_2,GPCR_3,MAPK_3,MEK_RAS_2,Metabolic_OR_3,PI3KAKT_4,Ribosomal_Activity_4,RNA_splicing_4,Ubiquitin_3,Wnt_signaling_1 |
| 7 | 3 | Cytoskeletal_5,Metabolic_FAS_3,PKC_3 |
| 8 | 12 | Cell_Cycle_3,Cytoskeletal_1,Heatshock_3,Histone2_1,Metabolic_Lipid_3,Metabolic_OR_1,Metabolic_OR_2,Metabolic_Protein_2,PKC_4,SMAD_1,STAT_4,Transcription_4 |
| 9 | 7 | Adhesion_2,GPCR_2,Hypoxia_2,MAPK_4,Phosphatase_1,SRC_1,T_Cell_1 |
| 10 | 17 | Apoptosis_BH3_2,Apoptosis_Occuring_4,Apoptosis_Regulating_3,Cell_Cycle_5,Cytoskeletal_2,Hippo_3,Histone1_2,MAPK_2,MEK_RAS_1,MTOR_3,PI3KAKT_2,Ribosomal_Activity_1,RNA_splicing_3,STAT_1,TP53_1,Transcription_3,Wnt_signaling_2 |
| 11 | 11 | Differentiation_1,Heatshock_2,Metabolic_FAS_4,Metabolic_Lipid_2,Metabolic_OR_4,PKC_2,SMAD_2,SMAD_3,SRC_2,STP_Regulation_1,T_Cell_2 |
| 12 | 6 | Apoptosis_Occuring_2,CREB_1,Cytoskeletal_3,Phosphatase_3,PKC_5,Ubiquitin_2 |
| 13 | 18 | Adhesion_1,Autophagy_1,BCR_1,BCR_3,CREB_2,Differentiation_3,DNA_damage_3,Heatshock_1,Histone2_3,Metabolic_FAS_1,Metabolic_Glucose_2,Metabolic_Lipid_1,Metabolic_Protein_3,Ribosomal_Activity_2,STP_Regulation_2,Ubiquitin_1,UPR_1,UPR_3 |
